# Supplementary material for: Reprogramming of profibrotic macrophages for treatment of bleomycin‐induced pulmonary fibrosis
Source: EMBO Mol Med. 2020 Jun 29;12(8):e12034. doi: 10.15252/emmm.202012034 (PMC7411553; doi:10.15252/emmm.202012034)
Supplement: Supplementary file 1 — Appendix [file EMMM-12-e12034-s001.pdf]

Appendix

Content:

Appendix Fig. S1: Synthesis of FA-TLR7-54.

Appendix Fig. S2: Characterization of the structure of FA-TLR7-54.

Appendix Fig. S3: Treatment with nontargeted TLR7 agonist is sufficient to promote acquisition of an M1-like phenotype in murine bone marrow-derived macrophages (BMDMs).

Appendix Fig. S4: Demonstration that THP-1 cells express FRβ following induction with IL-4, IL-6 plus IL-13 similar to naturally occurring IPF lung macrophages.

Appendix Table S1: PCR primer sequences

Appendix Table S2: List of exact *P*-values

Appendix materials and methods

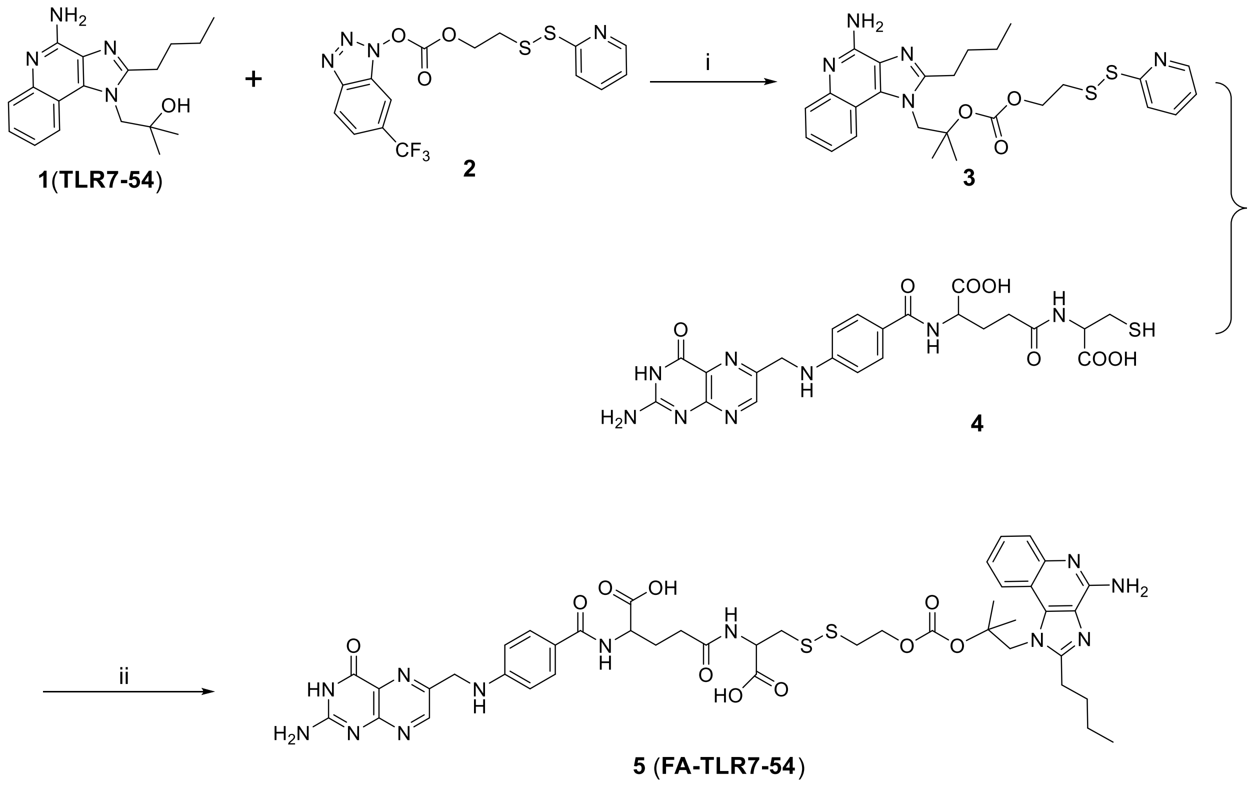

Appendix Fig. S1. Synthesis of FA-TLR7-54. Reagents and conditions: (i) DMAP, methylene chloride, argon atmosphere, room temperature; (ii) DMAP, dimethyl sulfoxide, argon atmosphere, room temperature.

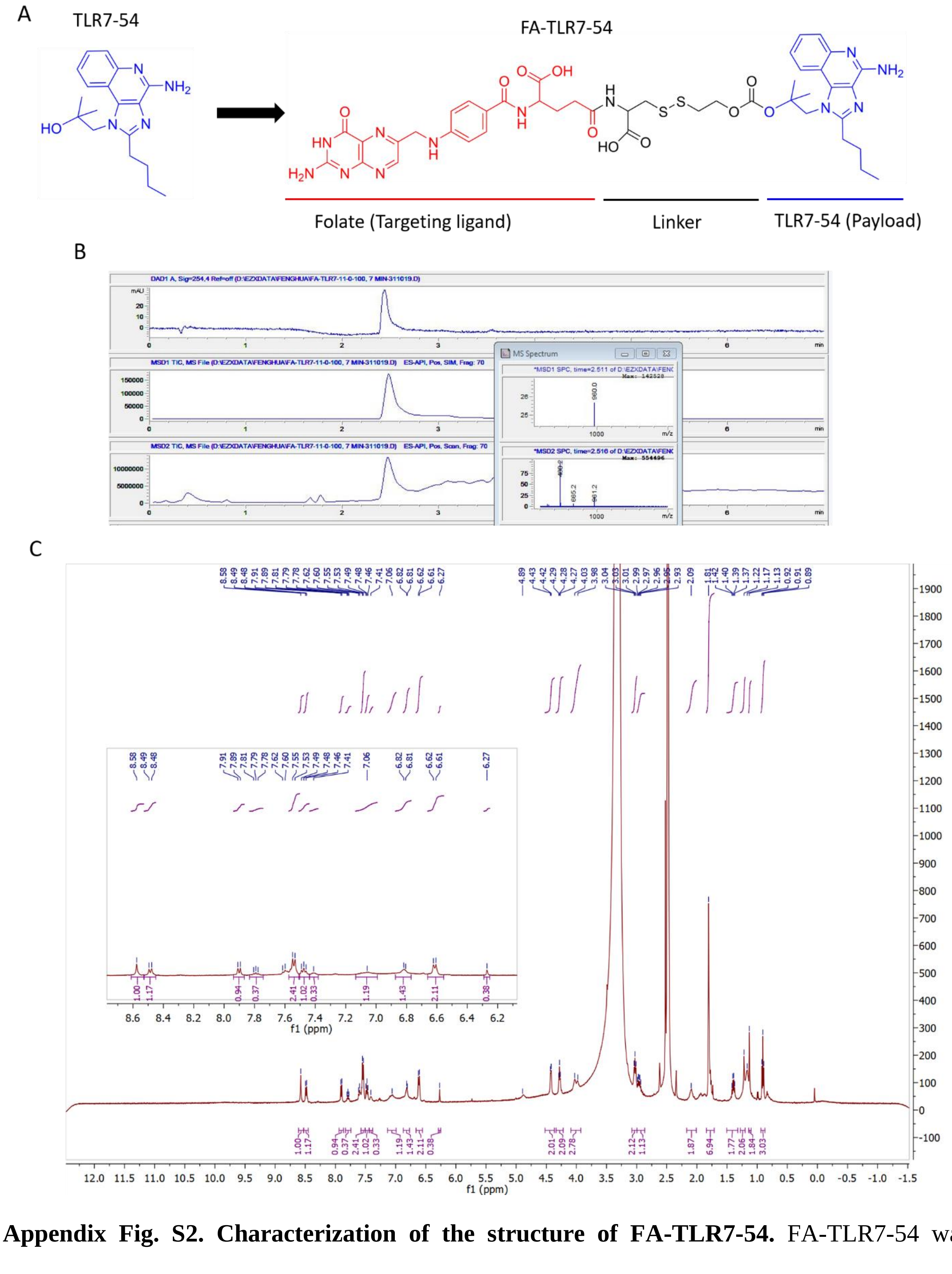

Appendix Fig. S2. Characterization of the structure of FA-TLR7-54. FA-TLR7-54 was constructed by conjugating folate (targeting ligand) with TLR7-54 (payload) via a self-immolative

linker (Vlahov & Leamon, 2012) (**A**). LC-MS spectrum (**B**) and ^{1}H NMR spectrum (**C**) of FA-TLR7-54.

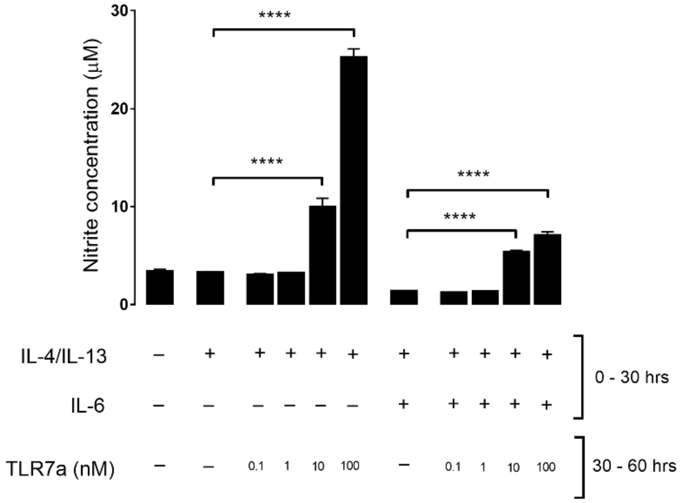

Appendix Fig. S3. Treatment with nontargeted TLR7 agonist is sufficient to promote acquisition of an M1-like phenotype in murine bone marrow-derived macrophages (BMDMs). Mouse BMDMs were stimulated with IL-4, IL-13, and/or IL-6 to develop a polarized M2-like phenotype, after which cells were treated with the indicated concentrations of TLR7-54 for 30 hours. The supernatant was then collected, and nitrite was quantified as an indicator of nitric oxide production by M1-like macrophages. Each value represents the mean ± S.D. for each group. ****P<0.001 (TLR7-54 treated groups versus M2-untreated group compared using unpaired two-tailed t-test).

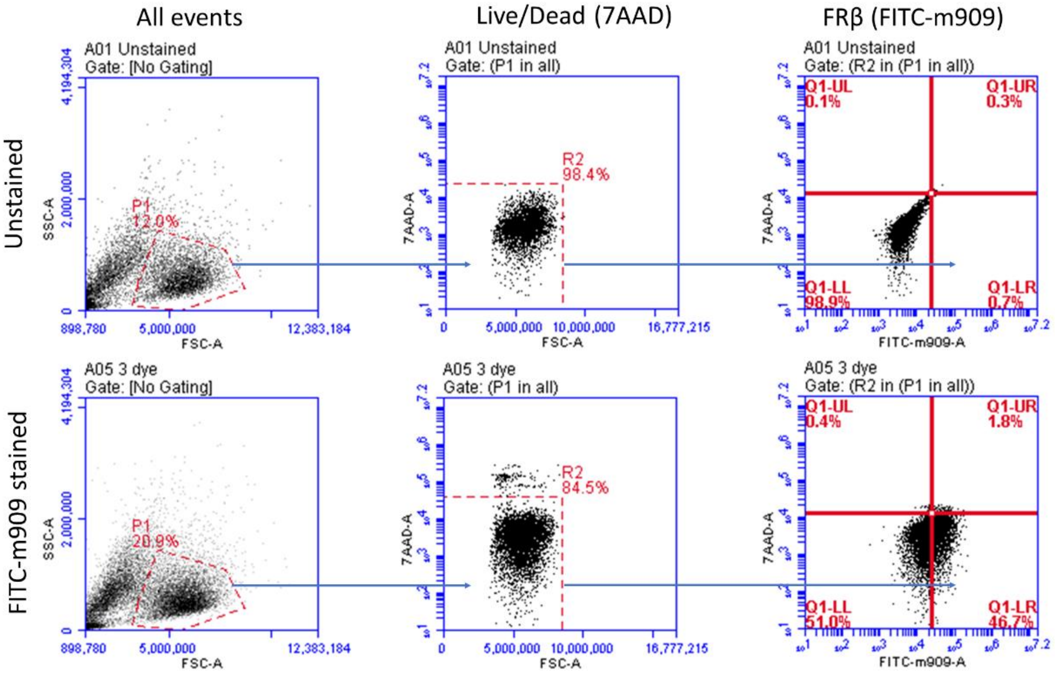

Appendix Fig. S4. Demonstration that THP-1 cells express FRβ following induction with IL-4, IL-6 plus IL-13 similar to naturally occurring IPF lung macrophages. THP-1 cells induced with IL-4, IL-6 plus IL-13 were either left unstained (upper panel) or stained with FITC-labeled monoclonal antibody (m909) to human FRβ (lower panel) prior to flow cytometry. Flow cytometry plots reveal the macrophage region defined by light scatter (P1), live cells defined as 7AAD- (R2), and FRβ$^+$ cells (Q1-LR).

Appendix Table S1. PCR primer sequences

Human CCL18	forward	5'-TGACTATTCTGAAACCAGCCC-3'
	reverse	5'-AGGTCGCTGATGTATTTCTGG-3'
Human CD206	forward	5'-GAGGTACACTAACTGGGCTG-3'
	reverse	5'-GGGTTCAGTAGCAGGGATTT-3'
Human IL-1β	forward	5'-GACAAAATACCTGTGGCCTTG-3'
	reverse	5'-AGACAAATCGCTTTTCCATCTTC-3'
Human Arg1	forward	5'-TTGATGTTGACGGACTGGAC-3'
	reverse	5'-CCTGAGAGTAGCCCTGTTTTG-3'
Human CD163	forward	5'-GGATTGTCCTGCCAGACG-3'
	reverse	5'-TCCCGACTGCAATAAAGGATG-3'
Human GAPDH	forward	5'-ACAACTTTGGTATCGTGGAAGG-3'
	reverse	5'-GCCATCACGCCACAGTTTC-3'
Human RPS9	forward	5'-GGATTTCTTAGAGAGACGCCTG-3'
	reverse	5'-GGACAATGAAGGACGGGATG-3'
Murine Arg1	forward	5'-AAGAATGGAAGAGTCAGTGTGG-3'
	reverse	5'-GGGAGTGTTGATGTCAGTGTG-3'
Murine CD206	forward	5'-ATGGATGTTGATGGCTACTGG-3'
	reverse	5'-TTCTGACTCTGGACACTTGC-3'
Murine CD163	forward	5'-AGTCATCTGCACTGGGAAAG-3'
	reverse	5'-CAGTTTTCTTTGTGGGCTTCG-3'
Murine CXCL10	forward	5'-TCAGCACCATGAACCCAAG-3'
	reverse	5'-CTATGGCCCTCATTCTCACTG-3'
Murine IL-6	forward	5'-CAAAGCCAGAGTCCTTCAGAG-3'
	reverse	5'-GTCCTTAGCCACTCCTTCTG-3'
Murine TNFα	forward	5'-CTTCTGTCTACTGAACTTCGGG-3'
	reverse	5'-CAGGCTTGTCACTCGAATTTTG-3'
Murine MMP9	forward	5'-GATCCCCAGAGCGTCATTC-3'
	reverse	5'-CCACCTTGTTCACCTCATTTTG-3'
Murine TIMP3	forward	5'-TGAAGGCAAGATGTACACAGG-3'
	reverse	5'-GAGGTCACAAAACAAGGCAAG-3'
Murine CD86	forward	5'-GAGTTTCCATCTGCTCAAACG-3'
	reverse	5'-ACTTAGAGGCTGTGTTGCTG-3'
Murine IRAK4	forward	5'-CTGTGGATGAAAACCGTGAAC-3'
	reverse	5'-AGAGTACATTGCTTCCACCG-3'
Murine β-Actin	forward	5'-ACCTTCTACAATGAGCTGCG-3'
	reverse	5'-CTGGATGGCTACGTACATGG-3'

Appendix Table S2. List of exact *P*-values

Figure	Panel	sub-panel	compared groups, *P*-value
Fig. 1	A	CCL18	TLR7-54, 0.1 nM vs 0 nM <0.0001<br>TLR7-54, 1 nM vs 0 nM <0.0001<br>TLR7-54, 10 nM vs 0 nM <0.0001<br>FA-TLR7-54, 0.1 nM vs 0 nM <0.0001<br>FA-TLR7-54, 1 nM vs 0 nM <0.0001<br>FA-TLR7-54, 10 nM vs 0 nM <0.0001
		IL-1β	TLR7-54, 0.1 nM vs 0 nM <0.0001<br>TLR7-54, 1 nM vs 0 nM <0.0001<br>TLR7-54, 10 nM vs 0 nM <0.0001<br>FA-TLR7-54, 0.1 nM vs 0 nM <0.0001<br>FA-TLR7-54, 1 nM vs 0 nM <0.0001<br>FA-TLR7-54, 10 nM vs 0 nM <0.0001
		CD206	TLR7-54, 0.1 nM vs 0 nM <0.0001<br>TLR7-54, 1 nM vs 0 nM <0.0001<br>TLR7-54, 10 nM vs 0 nM <0.0001<br>FA-TLR7-54, 0.1 nM vs 0 nM 0.8470<br>FA-TLR7-54, 1 nM vs 0 nM 0.0007<br>FA-TLR7-54, 10 nM vs 0 nM 0.0002
	B	CCL18	TLR7-54, 0.1 nM vs 0 nM <0.0001<br>TLR7-54, 1 nM vs 0 nM <0.0001<br>TLR7-54, 10 nM vs 0 nM 0.2470<br>FA-TLR7-54, 0.1 nM vs 0 nM <0.0001<br>FA-TLR7-54, 1 nM vs 0 nM <0.0001<br>FA-TLR7-54, 10 nM vs 0 nM <0.0001
		IL-1β	TLR7-54, 0.1 nM vs 0 nM 0.0003<br>TLR7-54, 1 nM vs 0 nM <0.0001<br>TLR7-54, 10 nM vs 0 nM <0.0001<br>FA-TLR7-54, 0.1 nM vs 0 nM 0.0002<br>FA-TLR7-54, 1 nM vs 0 nM 0.0003<br>FA-TLR7-54, 10 nM vs 0 nM <0.0001
	C	CCL18	TLR7-54, 0.1 nM vs 0 nM 0.0076<br>TLR7-54, 1 nM vs 0 nM 0.0024<br>TLR7-54, 10 nM vs 0 nM 0.0033<br>FA-TLR7-54, 0.1 nM vs 0 nM <0.0001<br>FA-TLR7-54, 1 nM vs 0 nM <0.0001<br>FA-TLR7-54, 10 nM vs 0 nM <0.0001
		IL-1β	TLR7-54, 0.1 nM vs 0 nM 0.7292<br>TLR7-54, 1 nM vs 0 nM 0.0234<br>TLR7-54, 10 nM vs 0 nM 0.6091<br>FA-TLR7-54, 0.1 nM vs 0 nM 0.9840<br>FA-TLR7-54, 1 nM vs 0 nM 0.0099<br>FA-TLR7-54, 10 nM vs 0 nM 0.0026
Fig. 2	A	48 h	TLR7-54 vs Untreated 0.0070

			FA-TLR7-54 vs Untreated 0.0096
		2+46 h	TLR7-54 vs Untreated 0.7161<br>FA-TLR7-54 vs Untreated 0.0455<br>Competition vs Untreated 0.5086
	B	48 h	TLR7-54 vs Untreated 0.0014<br>FA-TLR7-54 vs Untreated 0.0011
		2+46 h	TLR7-54 vs Untreated 0.0471<br>FA-TLR7-54 vs Untreated 0.0384<br>Competition vs Untreated 0.9715<br>FA-TLR7-54 vs Competition 0.0070
	C	48 h	TLR7-54 vs Untreated 0.0829<br>FA-TLR7-54 vs Untreated 0.0048
		2+46 h	TLR7-54 vs Untreated 0.1302<br>FA-TLR7-54 vs Untreated 0.0128<br>Competition vs Untreated 0.5982
	D	48 h	TLR7-54 vs Untreated 0.1701<br>FA-TLR7-54 vs Untreated 0.0273
		2+46 h	TLR7-54 vs Untreated 0.0256<br>FA-TLR7-54 vs Untreated 0.0220<br>Competition vs Untreated 0.4224<br>FA-TLR7-54 vs Competition 0.0371
	E	48 h	TLR7-54 vs Untreated 0.0474<br>FA-TLR7-54 vs Untreated 0.0438
		2+46 h	TLR7-54 vs Untreated 0.1866<br>FA-TLR7-54 vs Untreated 0.0343<br>Competition vs Untreated 0.9270<br>FA-TLR7-54 vs Competition 0.0341
	F	48 h	TLR7-54 vs Untreated <0.0001<br>FA-TLR7-54 vs Untreated <0.0001
		2+46 h	TLR7-54 vs Untreated 0.2835<br>FA-TLR7-54 vs Untreated 0.0004<br>Competition vs Untreated 0.1271<br>FA-TLR7-54 vs Competition 0.0003
Fig. 3	C		OTL38 vs Vehicle 0.3763<br>OTL38 vs OTL38+competition 0.6183
	D		OTL38 vs Vehicle <0.0001<br>OTL38 vs OTL38+competition 0.0002
Fig. 4	A	Arg1	Healthy vs Vehicle <0.0001<br>TLR7-54 vs Vehicle 0.0007<br>FA-TLR7-54 vs Vehicle 0.0003<br>TLR7-54 vs FA-TLR7-54 0.0030
		CD206	Healthy vs Vehicle 0.0006<br>TLR7-54 vs Vehicle 0.0024<br>FA-TLR7-54 vs Vehicle 0.0045<br>TLR7-54 vs FA-TLR7-54 0.0247

		CD163	Healthy vs Vehicle 0.1520<br>TLR7-54 vs Vehicle 0.0416<br>FA-TLR7-54 vs Vehicle 0.0030<br>TLR7-54 vs FA-TLR7-54 0.0633
		CXCL10	Healthy vs Vehicle 0.0150<br>TLR7-54 vs Vehicle 0.0002<br>FA-TLR7-54 vs Vehicle <0.0001<br>TLR7-54 vs FA-TLR7-54 0.0004
		IL-6	Healthy vs Vehicle 0.0013<br>TLR7-54 vs Vehicle <0.0001<br>FA-TLR7-54 vs Vehicle <0.0001<br>TLR7-54 vs FA-TLR7-54 0.5255
		TNFα	Healthy vs Vehicle 0.0037<br>TLR7-54 vs Vehicle <0.0001<br>FA-TLR7-54 vs Vehicle 0.0002<br>TLR7-54 vs FA-TLR7-54 <0.0001
	B	Arg1	Healthy vs Vehicle 0.0074<br>TLR7-54 vs Vehicle 0.0055<br>FA-TLR7-54 vs Vehicle 0.0061<br>TLR7-54 vs FA-TLR7-54 0.3154
		CD206	Healthy vs Vehicle <0.0001<br>TLR7-54 vs Vehicle <0.0001<br>FA-TLR7-54 vs Vehicle <0.0001<br>TLR7-54 vs FA-TLR7-54 0.0016
		CD163	Healthy vs Vehicle 0.0007<br>TLR7-54 vs Vehicle 0.0294<br>FA-TLR7-54 vs Vehicle 0.0215<br>TLR7-54 vs FA-TLR7-54 0.9705
		CXCL10	Healthy vs Vehicle 0.2645<br>TLR7-54 vs Vehicle 0.0264<br>FA-TLR7-54 vs Vehicle 0.0494<br>TLR7-54 vs FA-TLR7-54 0.8677
		IL-6	Healthy vs Vehicle 0.1865<br>TLR7-54 vs Vehicle 0.0001<br>FA-TLR7-54 vs Vehicle 0.1323<br>TLR7-54 vs FA-TLR7-54 0.0002
		TNFα	Healthy vs Vehicle 0.0059<br>TLR7-54 vs Vehicle 0.0002<br>FA-TLR7-54 vs Vehicle 0.0021<br>TLR7-54 vs FA-TLR7-54 0.0090
	C	IL-6	1h Healthy vs Vehicle 0.3992<br>1h TLR7-54 vs Vehicle <0.0001<br>1h FA-TLR7-54 vs Vehicle >0.9999<br>4h Healthy vs Vehicle 0.3332<br>4h TLR7-54 vs Vehicle <0.0001<br>4h FA-TLR7-54 vs Vehicle <0.0001

		IFNα	1h Healthy vs Vehicle 0.1487<br>1h TLR7-54 vs Vehicle 0.0012<br>1h FA-TLR7-54 vs Vehicle 0.0047<br>4h Healthy vs Vehicle 0.1774<br>4h TLR7-54 vs Vehicle 0.0121<br>4h FA-TLR7-54 vs Vehicle 0.0198
		TNFα	1h Healthy vs Vehicle 0.9985<br>1h TLR7-54 vs Vehicle <0.0001<br>1h FA-TLR7-54 vs Vehicle 0.3301<br>4h Healthy vs Vehicle 0.9992<br>4h TLR7-54 vs Vehicle 0.2679<br>4h FA-TLR7-54 vs Vehicle 0.9454
Fig. 5	C		Healthy vs Vehicle 0.0002<br>FA-TLR7-54 vs Vehicle 0.0048
	D		Healthy vs Vehicle 0.0362<br>FA-TLR7-54 vs Vehicle 0.0269
	E		Healthy vs Vehicle 0.0064<br>FA-TLR7-54 vs Vehicle 0.0171
	F		Healthy vs Vehicle 0.0007<br>FA-TLR7-54 vs Vehicle 0.0027
	G		Healthy vs Vehicle 0.2372<br>FA-TLR7-54 vs Vehicle 0.0020
	H		Healthy vs Vehicle 0.0002<br>FA-TLR7-54 vs Vehicle 0.0023
	J		Healthy vs Vehicle 0.0001<br>FA-TLR7-54 vs Vehicle 0.0136
	K		Healthy vs Vehicle 0.0002<br>FA-TLR7-54 vs Vehicle 0.0486
Fig. 6	B		Healthy vs Vehicle 0.0018<br>FA-TLR7-54, 1 nmol vs Vehicle 0.2177<br>FA-TLR7-54, 3 nmol vs Vehicle 0.1700<br>FA-TLR7-54, 10 nmol vs Vehicle 0.0470
	C		Healthy vs Vehicle 0.0014<br>FA-TLR7-54, 1 nmol vs Vehicle 0.4051<br>FA-TLR7-54, 3 nmol vs Vehicle 0.1769<br>FA-TLR7-54, 10 nmol vs Vehicle 0.0264
Fig. 7	D		TLR7-54 10 nmol vs Vehicle 0.0067<br>FA-TLR7-54 3 nmol vs TLR7-54 10 nmol 0.0067<br>FA-TLR7-54 10 nmol vs TLR7-54 10 nmol 0.0079<br>FA-TLR7-54 20 nmol vs TLR7-54 10 nmol 0.0203
	E		TLR7-54 10 nmol vs Vehicle 0.0009<br>FA-TLR7-54 3 nmol vs TLR7-54 10 nmol 0.0009<br>FA-TLR7-54 10 nmol vs TLR7-54 10 nmol 0.0010<br>FA-TLR7-54 20 nmol vs TLR7-54 10 nmol 0.0020
	F		TLR7-54 10 nmol vs Vehicle 0.0425<br>FA-TLR7-54 3 nmol vs TLR7-54 10 nmol 0.0425

			FA-TLR7-54 10 nmol vs TLR7-54 10 nmol 0.0450<br>FA-TLR7-54 20 nmol vs TLR7-54 10 nmol 0.1319
Fig. EV2		THP1/NF-κB-luc	TLR7-54 100 nM vs Untreated 0.1234
		THP1/NF-κB-luc/hTLR7	TLR7-54 100nM vs Untreated 0.0005<br>TNFα 10ng/ml vs Untreated 0.0058
Fig. EV3	C		IMs Bleo vs control 0.2706<br>Mono-AMs Bleo vs control <0.0001<br>TR-AMs Bleo vs control <0.0001
	D		FRβ$^+$ IMs Bleo vs control 0.2668<br>FRβ$^+$ Mono-AMs Bleo vs control 0.0089<br>FRβ$^+$ Mono-AMs Bleo vs control 0.8286
Appendix Fig. S3		IL-4/IL-13	TLR7-54 0.1 nM vs Vehicle 0.9993<br>TLR7-54 1 nM vs Vehicle >0.9999<br>TLR7-54 10 nM vs Vehicle <0.0001<br>TLR7-54 100 nM vs Vehicle <0.0001
		IL-4/IL-13/IL-6	TLR7-54 0.1 nM vs Vehicle 0.9827<br>TLR7-54 1 nM vs Vehicle >0.9999<br>TLR7-54 10 nM vs Vehicle <0.0001<br>TLR7-54 100 nM vs Vehicle <0.0001

Appendix materials and methods

Synthesis of FA-TLR7-54

TLR7-54 (**1**) and the heterobifunctional disulfide linker (**2**) were synthesized as described elsewhere (Satyam, 2008; Shukla et al, 2010) and reacted in the presence of dimethylaminopyridine (DMAP) in methylene chloride to form the mixed disulfide (**3**). Folate-cysteine (**4**) was prepared as described previously (Kularatne & Low, 2010) and then reacted with **3** in dimethyl sulfoxide with DMAP to afford the final product FA-TLR7-54 (**5**) (Appendix Fig S1). FA-TLR7-54 was purified by preparative HPLC and characterized by LC/MS and ^{1}H NMR (see Appendix Fig S2, panels B and C). ^{1}H NMR (500 MHz, DMSO-d_6) δ 8.58 (s, 1H), 8.49 (d, J = 8.8 Hz, 1H), 7.90 (d, J = 8.3 Hz, 1H), 7.83 – 7.74 (m, 1H), 7.54 (d, J = 8.0 Hz, 2H), 7.48 (t, J = 7.6 Hz, 1H), 7.41 (s, 1H), 7.06 (s, 1H), 6.81 (d, J = 6.2 Hz, 1H), 6.61 (d, J = 8.3 Hz, 2H), 6.27 (s, 1H), 4.43 (d, J = 5.9 Hz, 2H), 4.28 (t, J = 6.6 Hz, 2H), 4.00 (d, J = 25.7 Hz, 3H), 3.03 (t, J = 7.5

Hz, 2H), 2.97 (dd, *J* = 13.0, 6.5 Hz, 1H), 2.09 (s, 2H), 1.81 (s, 7H), 1.40 (q, *J* = 7.4 Hz, 2H), 1.22 (s, 2H), 1.13 (s, 2H), 0.91 (t, *J* = 7.4 Hz, 3H). MS (ESI) calculated for $C_{43}H_{50}N_{12}O_{10}S_2$, m/z 958.3, found 959.2 $(M + H)^+$.

Generation of stable THP-1/NF-κB-luc-GFP and THP-1/NF-κB-luc/hTLR7 reporter cells

THP-1 cells were transduced with a lentiviral vector containing an NF-κB-luc-GFP construct that stimulates expression of green fluorescent protein (GFP) and luciferase (luc) upon activation of NF-κB (System Bioscience). After selection in puromycin-containing media, a fraction of the cells was further transduced with lentiviral vector (pLV.Des2d.C/EGFP (Vector Builder)) to also express human TLR7 (hTLR7). After selection and expansion, these cells were used for following luciferase reporter assay.

Luciferase Reporter Assay

THP-1/NF-κB-luc-GFP and THP-1/NF-κB-luc/hTLR7 reporter cells described above were incubated in the absence or presence of 100 nM TLR7-54 or 10 ng/ml TNFα (positive control) for 6 h. Induced luciferase activity was then measured using the ONE-Glo™ Luciferase Assay System (Promega, E6110). Assays were performed in 96-well flat bottom plates at $1x10^5$ cells per well in a total volume of 100 µl (including stimulus).

Differentiation and polarization of Murine bone marrow derived macrophages (BMDMs)

Murine bone marrow cells were isolated from tibias and femurs of male C57BL/6 mice and cultured for 7 days in DMEM medium (plus 10% FBS and 1% Penicillin/streptomycin) containing 20 ng/ml recombinant mouse M-CSF (PeproTech). The resulting macrophages were then differentiated into M2-like macrophages by treatment for 30 hours in DMEM medium containing recombinant mouse 20 ng/ml IL-4 (Peprotech), 20 ng/ml IL-13 (Peprotech), alone, or in combination with 5ng/mL IL-6 (Peprotech).

Reference:

Kularatne SA, Low PS (2010) Targeting of Nanoparticles: Folate Receptor. In Cancer Nanotechnology: Methods and Protocols, Grobmyer SR, Moudgil BM (eds) pp 249-265. Totowa, NJ: Humana Press

Satyam A (2008) Design and synthesis of releasable folate–drug conjugates using a novel heterobifunctional disulfide-containing linker. Bioorg Med Chem Lett 18**:** 3196-3199

Shukla NM, Malladi SS, Mutz CA, Balakrishna R, David SA (2010) Structure−Activity Relationships in Human Toll-Like Receptor 7-Active Imidazoquinoline Analogues. J Med Chem 53**:** 4450-4465

Vlahov IR, Leamon CP (2012) Engineering Folate–Drug Conjugates to Target Cancer: From Chemistry to Clinic. Bioconjug Chem 23**:** 1357-1369
