# Supplementary material for: Reprogramming of profibrotic macrophages for treatment of bleomycin‐induced pulmonary fibrosis
Source: EMBO Mol Med. 2020 Jun 29;12(8):e12034. doi: 10.15252/emmm.202012034 (PMC7411553; doi:10.15252/emmm.202012034)
Supplement: Supplementary file 2 — Expanded View Figures PDF [file EMMM-12-e12034-s002.pdf]

Expanded View Figures

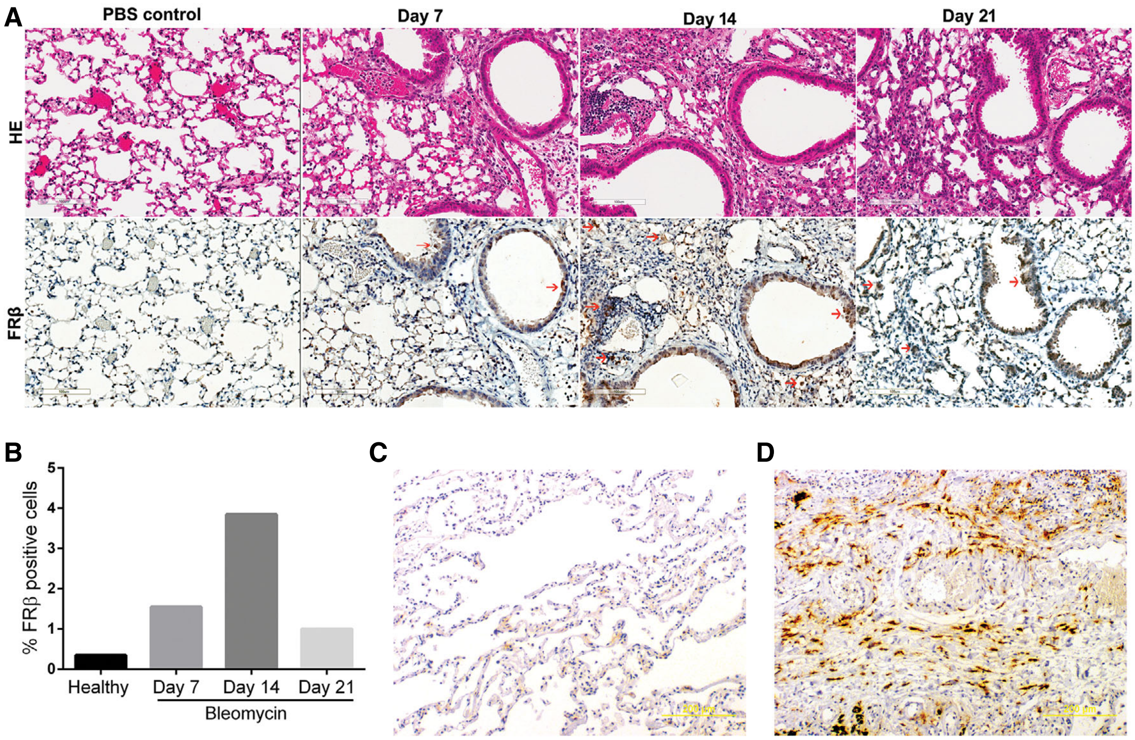

Figure EV1. FRβ expression in murine (A&B) and human (C&D) lungs.

A Mice with BLM-induced experimental fibrosis were stained using a monoclonal antibody to mouse FRβ (F3). Representative FRβ-positive macrophages are marked with red arrows. H&E and FRβ IHC staining were performed on days 7, 14, and 21 post-BLM-induced lung injury. More than 90×10^6 cells were quantified per section using Aperio Image Scope (Leica Biosystems). Scale bars, 100 μm.

B Quantification of FRβ staining in sections from panel A.

C, D IHC staining of healthy (C) or IPF (D) human lung tissue with a monoclonal antibody to human FRβ (m909). Scale bars, 200 μm.

Source data are available online for this figure.

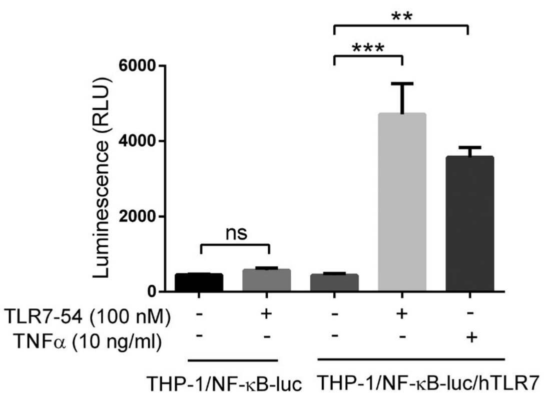

Figure EV2. TLR7-54 specifically targets TLR7.

NF-κB-luc-transduced or NF-κB-luc/hTLR7-transduced THP-1 cells were incubated with 100 nM TLR7-54 or 10 ng/ml TNFα (positive control) for 6 h. NF-κB-induced luciferase activity was then quantitated by ONE-Gloucodep™ Luciferase Assay (n = 3, technical replicates). Mean ± SD. Statistical significance between groups was determined using unpaired two-tailed t-test (**$P < 0.01$, ***$P < 0.001$).

 © 2020 The Authors

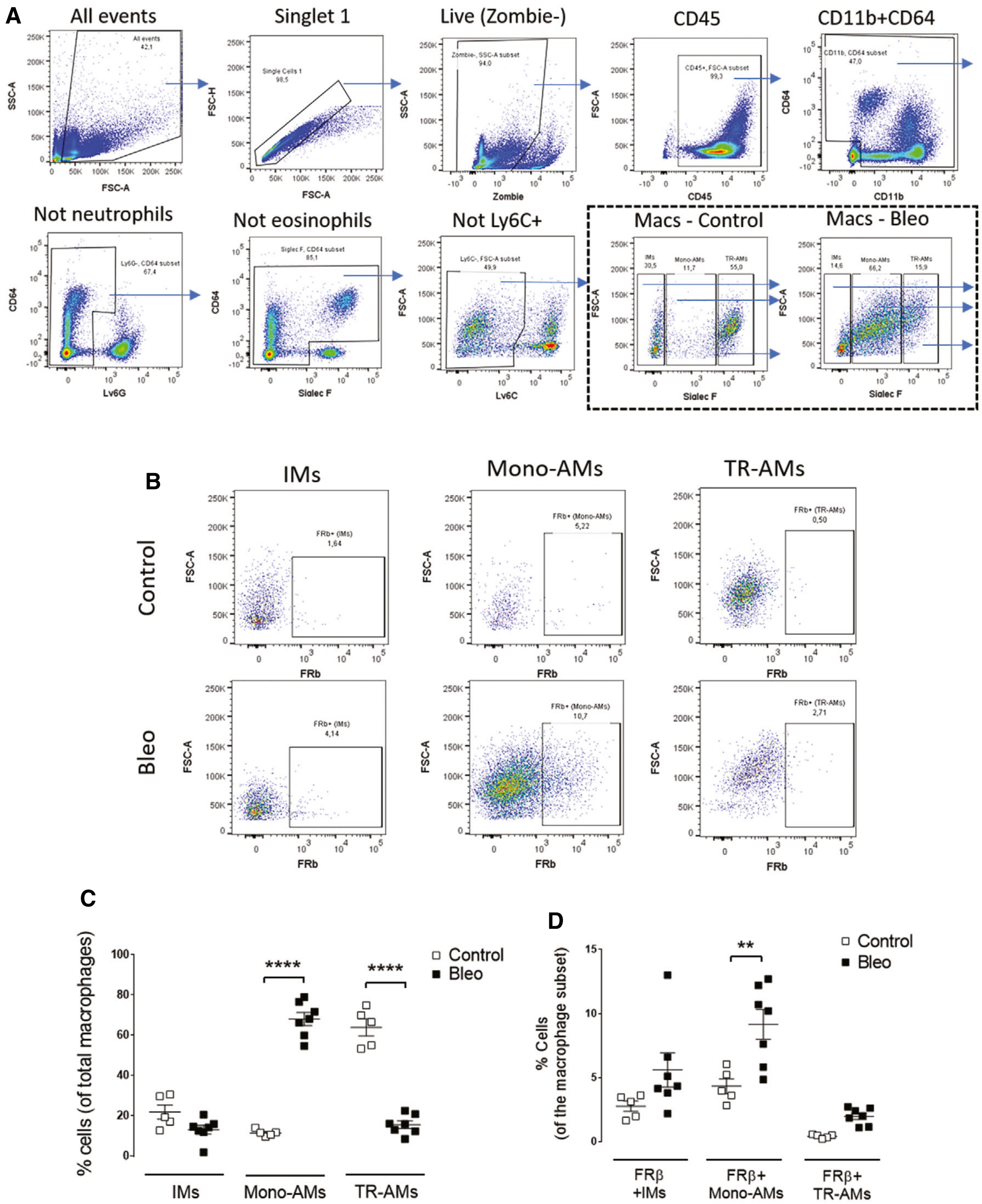
A
All events
Singlet 1
Live (Zombie-)
CD45
CD11b+CD64
Not neutrophils
Not eosinophils
Not Ly6C+
Macs - Control
Macs - Bleo
B
IMs
Mono-AMs
TR-AMs
Control
Bleo
C
D
Control
Bleo
% cells (of total macrophages)
% Cells (of the macrophage subset)
IMs
Mono-AMs
TR-AMs
FRβ +IMs
FRβ+ Mono-AMs
FRβ+ TR-AMs

Figure EV3.

Figure EV3. Monocyte-derived alveolar macrophages constitute the predominant macrophage subpopulation that expresses FRβ following BLM-induced lung injury.

Ten days following intratracheal instillation of bleomycin (0.75 mg/kg), mice were sacrificed and lungs were processed for flow cytometric staining and analysis.

- A Representative plots showing the gating strategy leading to various macrophage subpopulations.
- B Representative plots showing FRβ expression on interstitial macrophages (IMs), monocyte-derived alveolar macrophages (Mono-AMs), and tissue-resident alveolar macrophages (TR-AMs).
- C Percentages of IMs, Mono-AMs, and TR-AMs present in the total macrophage pool (Ly6C- gate) (n = 5–7).
- D Proportion of FRβ-expressing IMs, FRβ-expressing Mono-AMs, and FRβ-expressing TR-AMs in the corresponding parent populations (n = 5–7). All samples were derived at the same time and processed in parallel.

Data information: Mean ± SEM. Significance was compared with one-way ANOVA using Tukey's multiple comparison test (**P < 0.01, ****P < 0.0001).

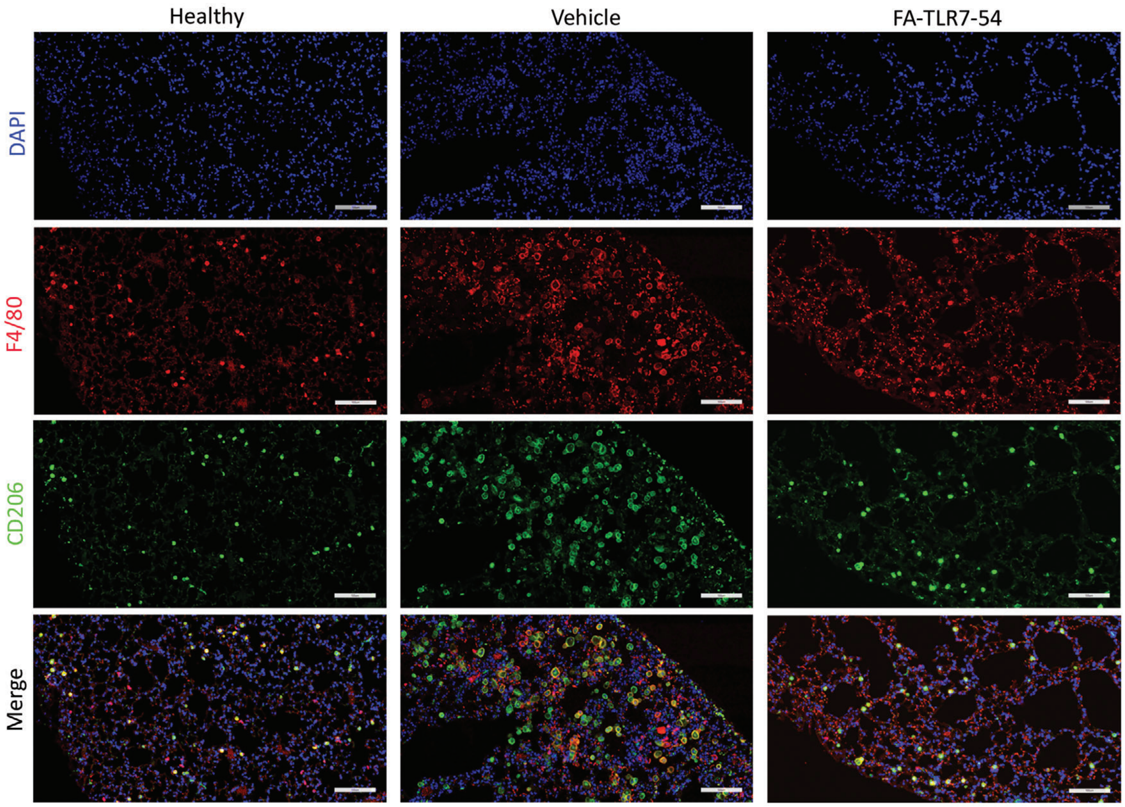

Figure EV4. Treatment with FA-TLR7-54 reduces CD206-positive macrophages in fibrotic lungs.

Sections from the same healthy and fibrotic lungs described in Fig 5 were stained with DAPI (nuclei; blue), anti-F4/80 (macrophages; red), and anti-CD206 (M2 macrophage marker; green), and images were obtained with a Leica Versa 8 whole-slide scanner as described in Materials and Methods (n = 2). Scale bars, 100 μm.
Source data are available online for this figure.

 © 2020 The Authors
