# Supplementary figures and images for: Reprogramming of profibrotic macrophages for treatment of bleomycin‐induced pulmonary fibrosis
Source: EMBO Mol Med. 2020 Jun 29;12(8):e12034. doi: 10.15252/emmm.202012034 (PMC7411553; doi:10.15252/emmm.202012034)

**Fig EV1. panel A**

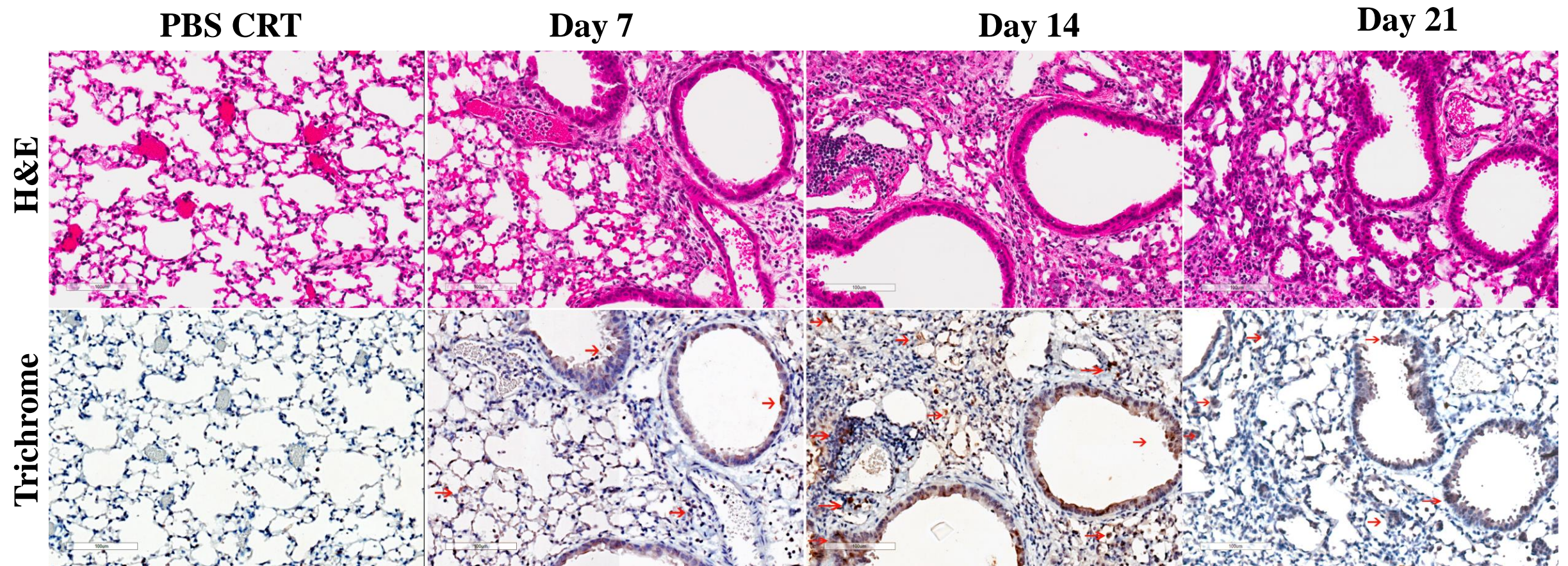

**Fig EV1.**

**panel C**

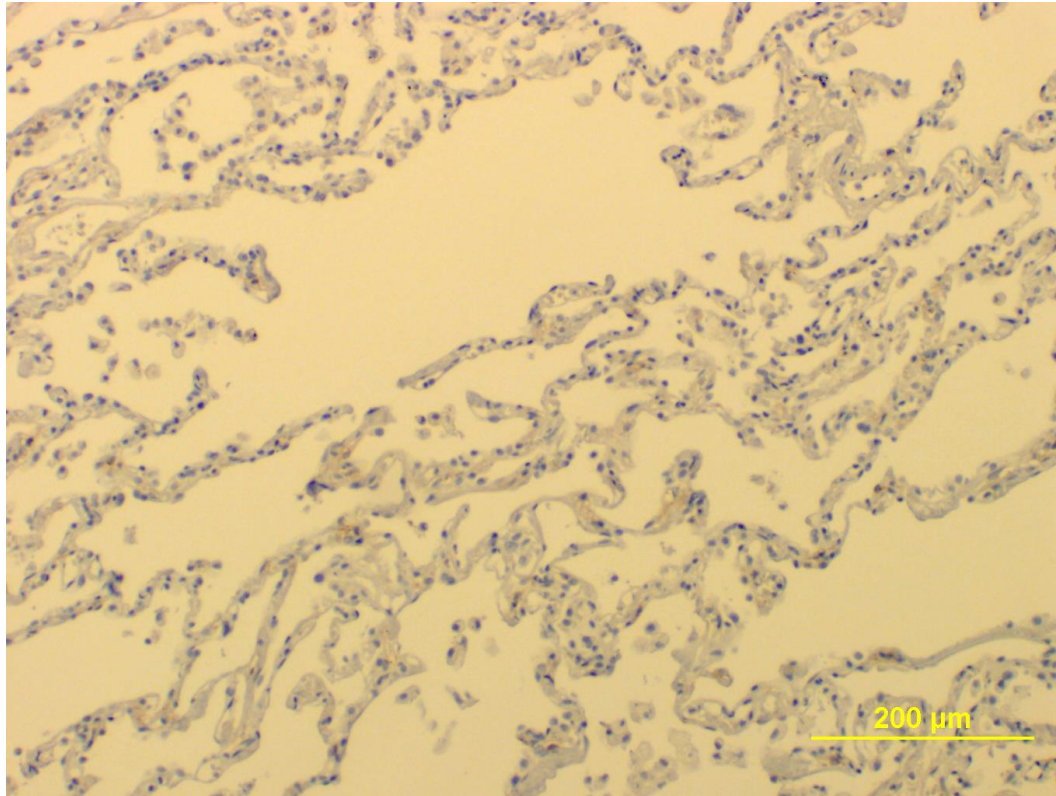

**panel D**

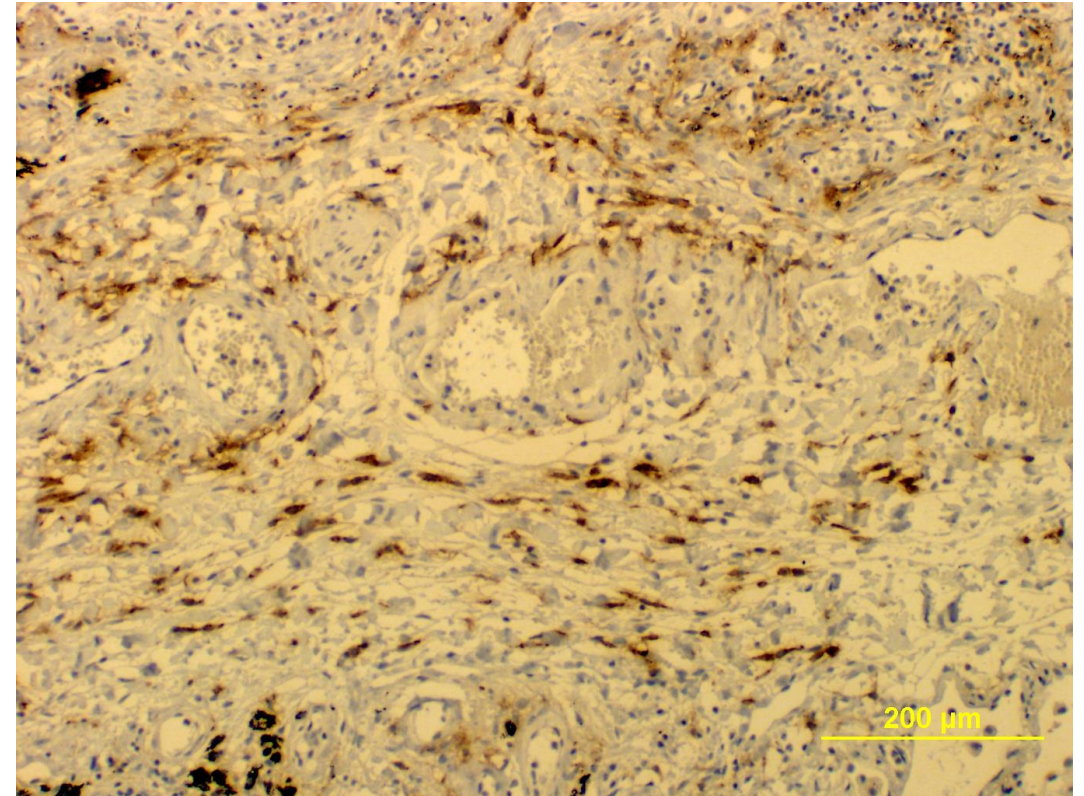

Supplement: Supplementary file 3 — Source Data for Expanded View [file EMMM-12-e12034-s006.zip › EV_source_data/EMM-2020-12034-V3-Figure_EV1_Source_Data-sd.pdf]

**Fig EV4.**

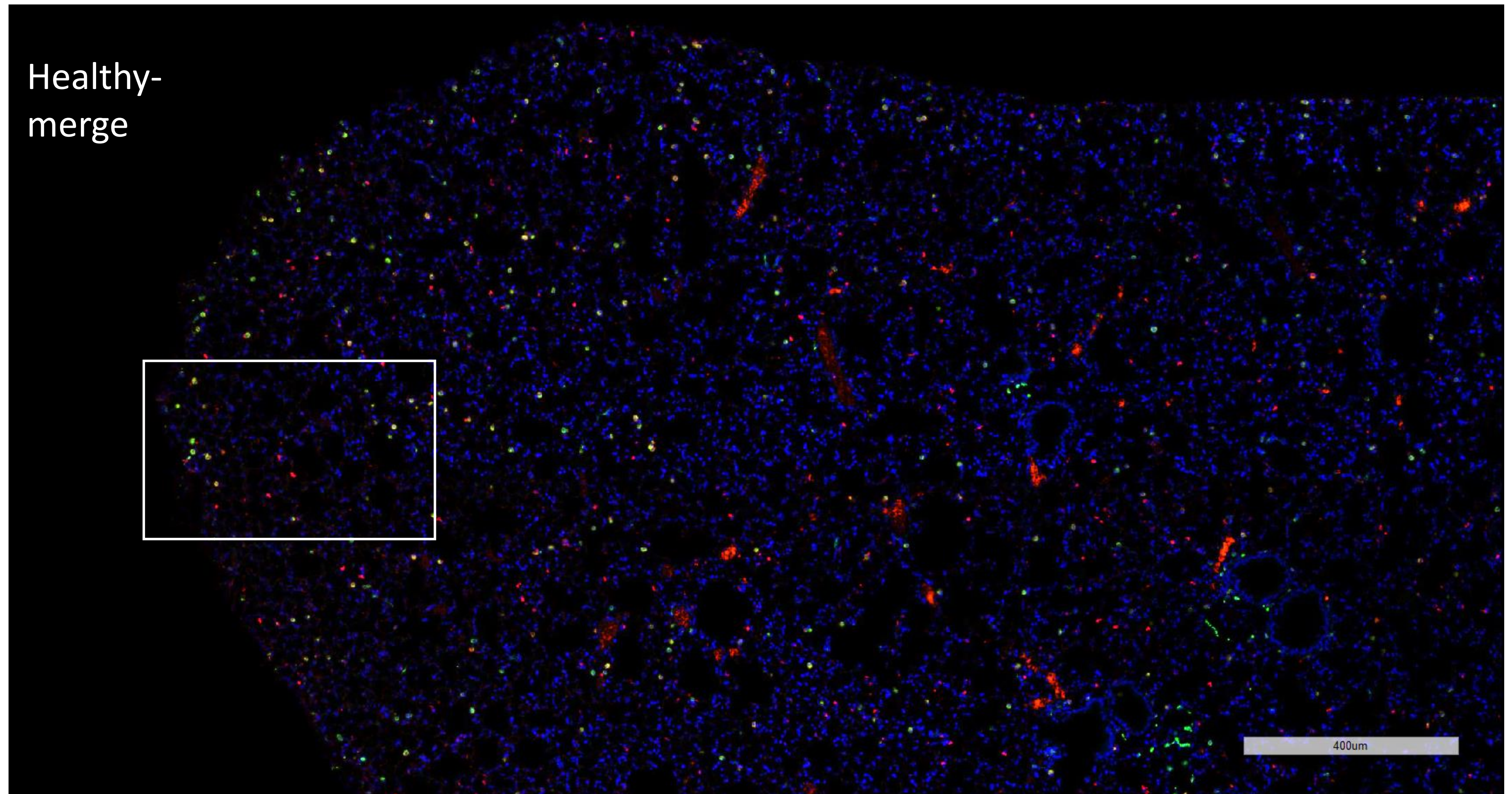

**Fig EV4.**

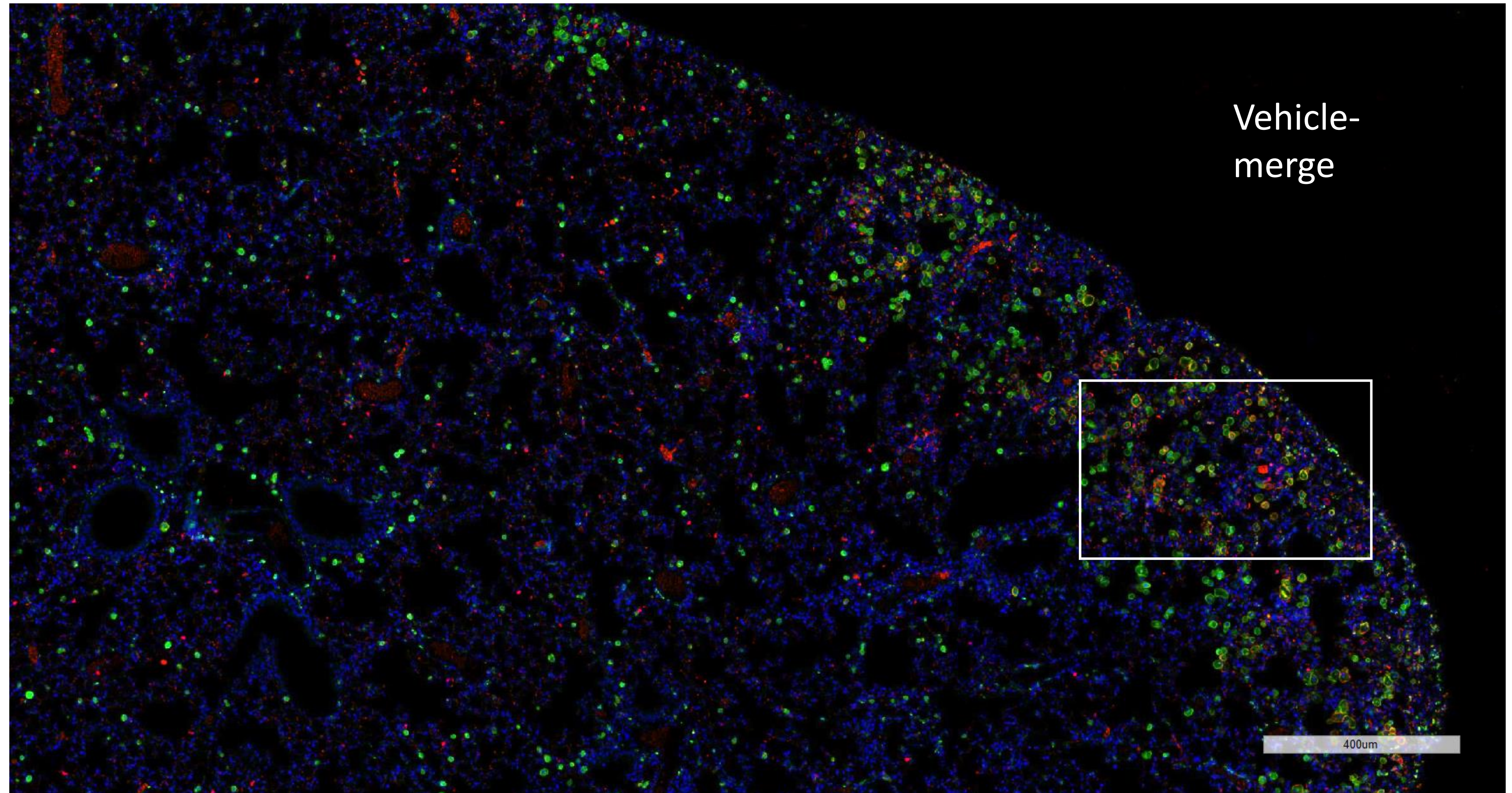

**Fig EV4.**

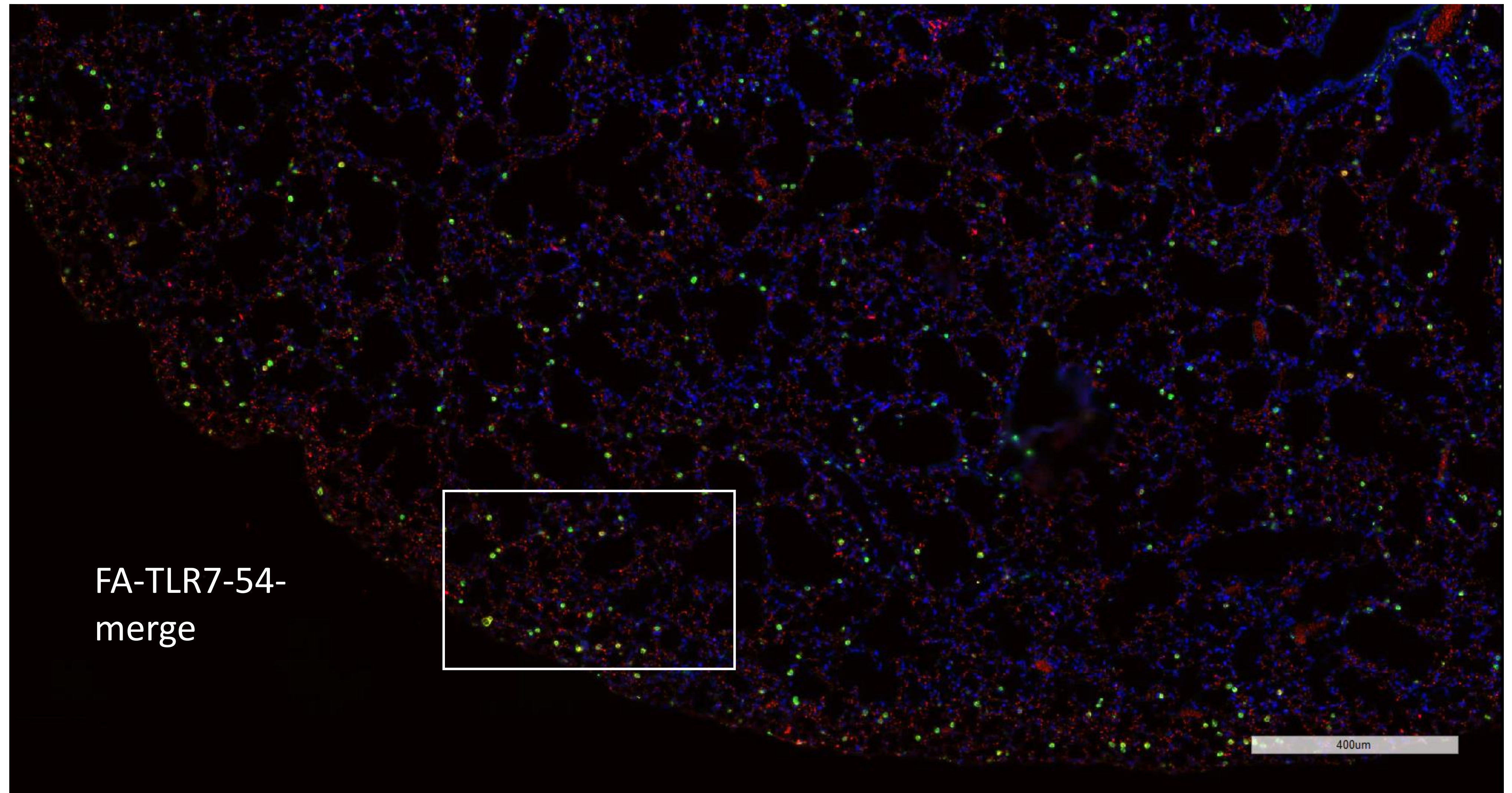

Supplement: Supplementary file 3 — Source Data for Expanded View [file EMMM-12-e12034-s006.zip › EV_source_data/EMM-2020-12034-V3-Figure_EV4_Source_Data-sd.pdf]

**Fig 3A**

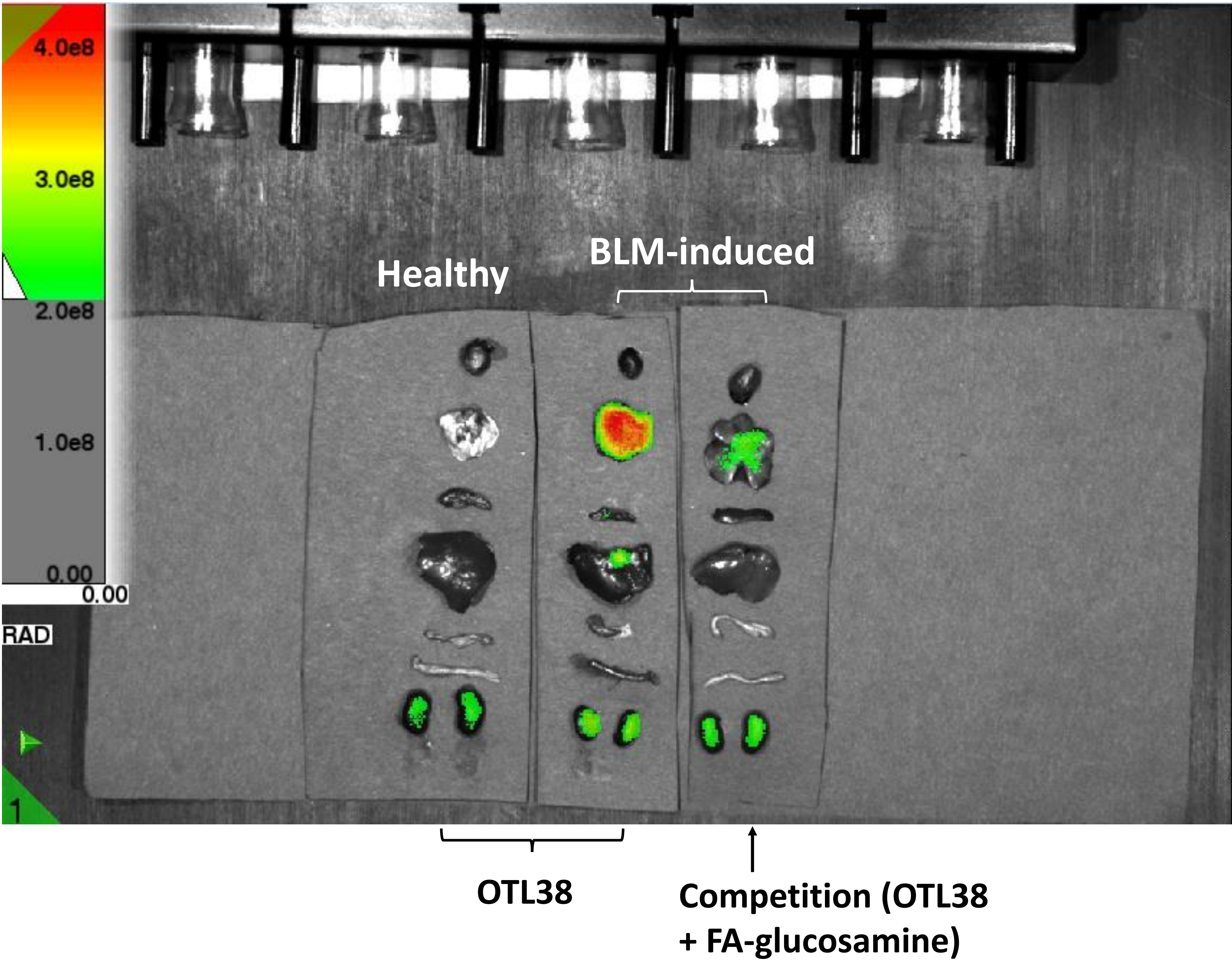

Supplement: Supplementary file 5 — Source Data for Figure 3 [file EMMM-12-e12034-s003.pdf]
