# Supplementary material for: Reprogramming of profibrotic macrophages for treatment of bleomycin‐induced pulmonary fibrosis
Source: EMBO Mol Med. 2020 Jun 29;12(8):e12034. doi: 10.15252/emmm.202012034 (PMC7411553; doi:10.15252/emmm.202012034)

Fig 5. panel I

H&E stains:

Healthy

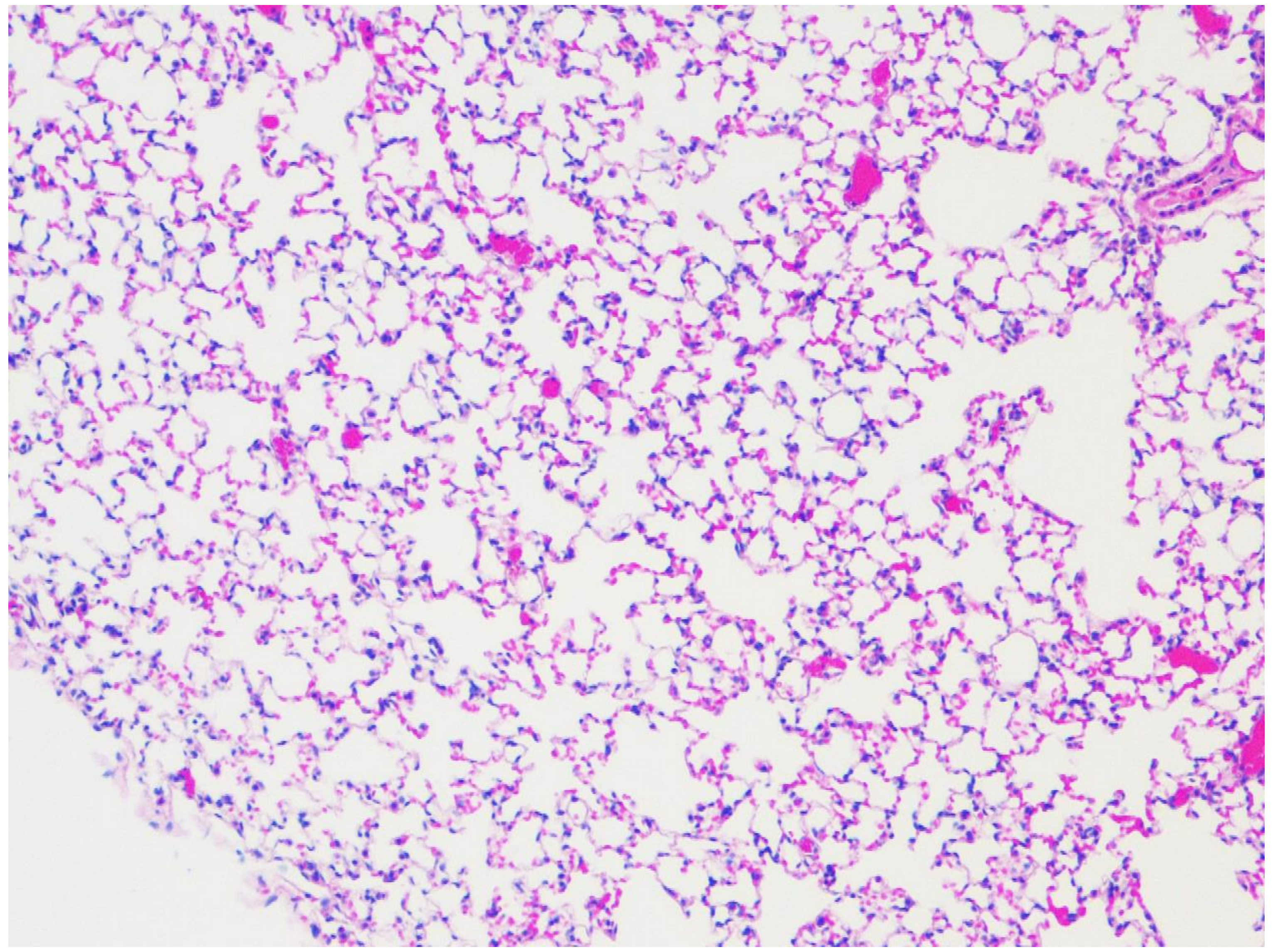

Vehicle

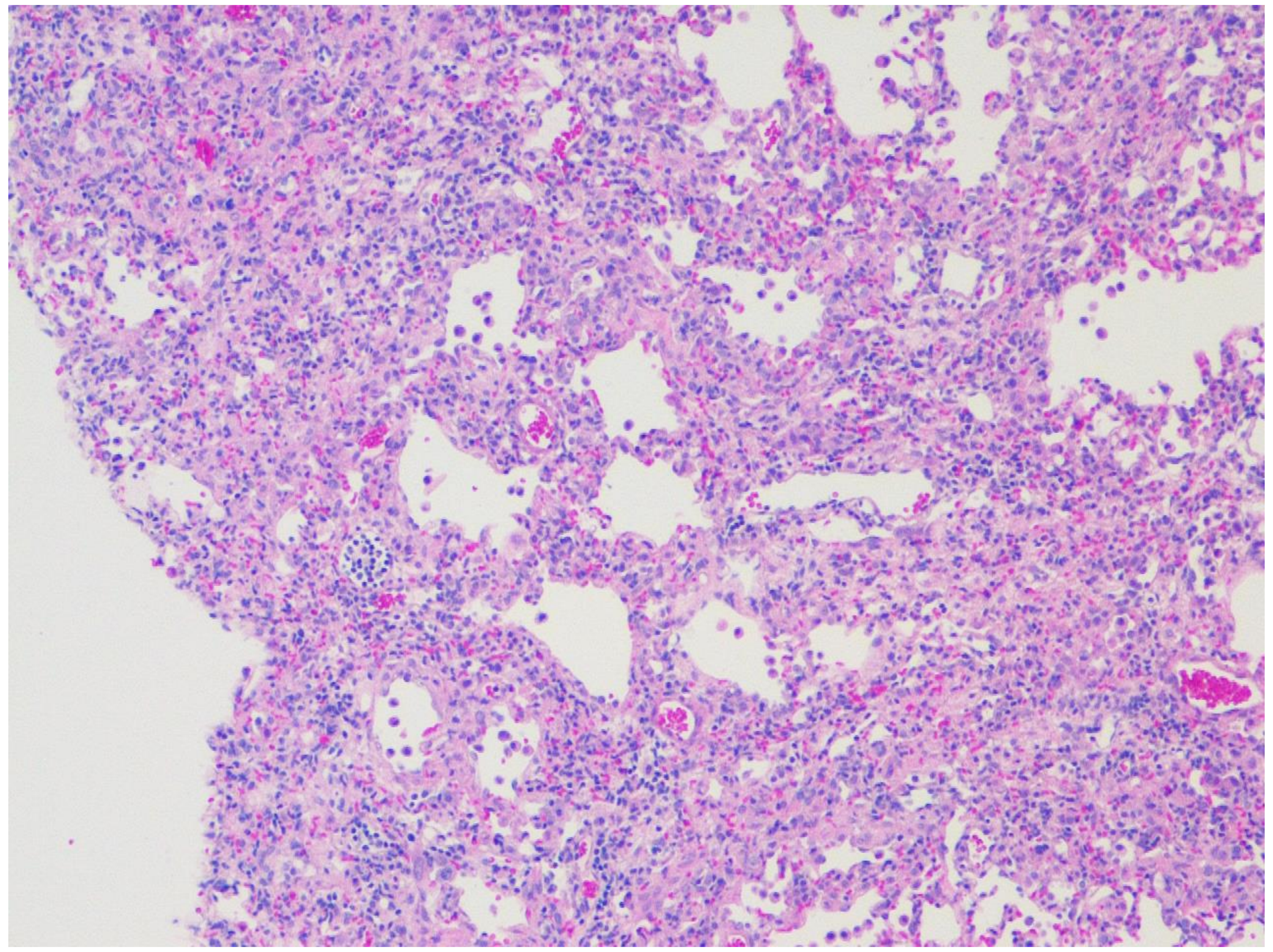

FA-TLR7-54

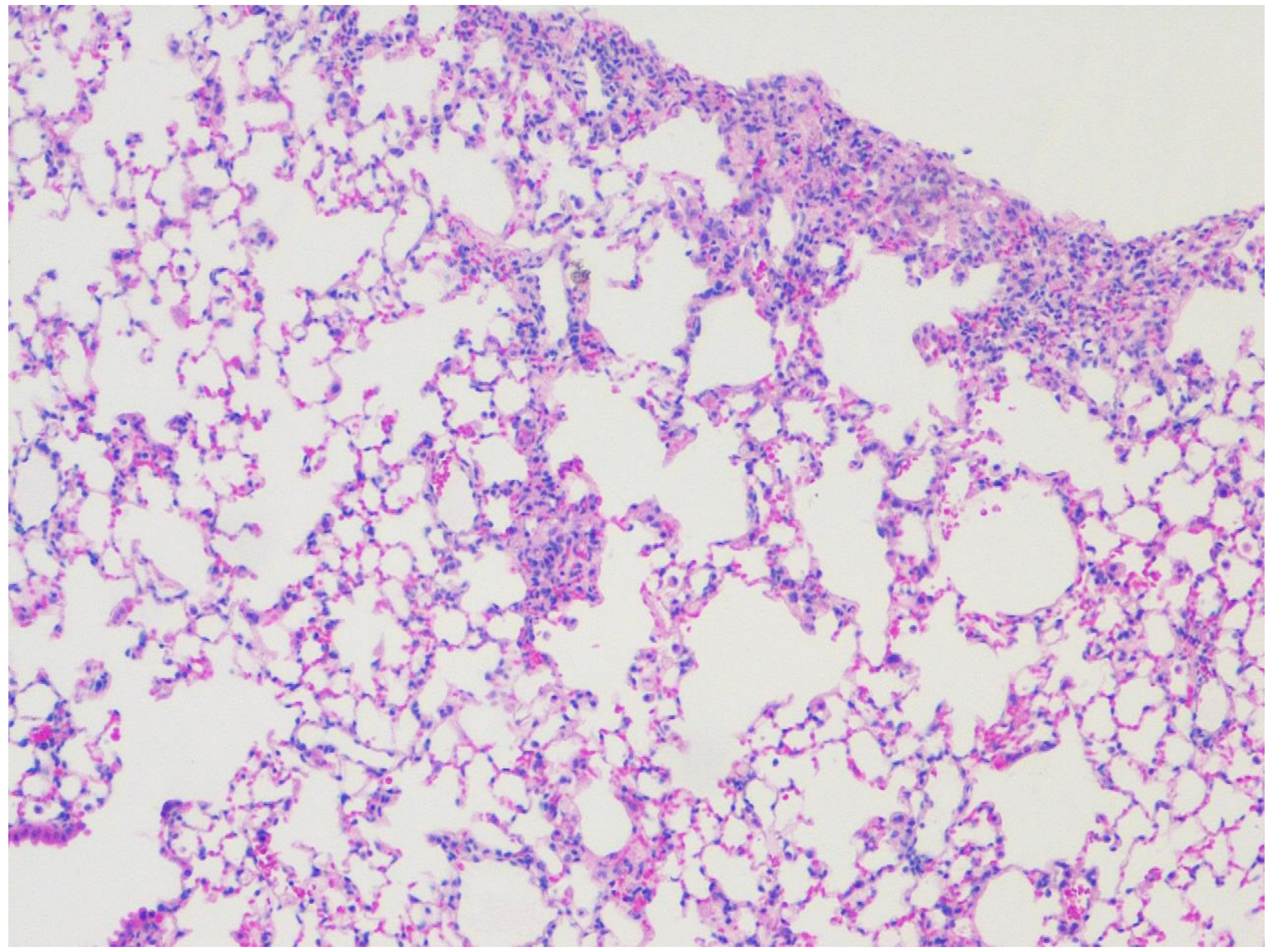

Fig 5. panel I

Trichrome stains:

Healthy Vehicle FA-TLR7-54

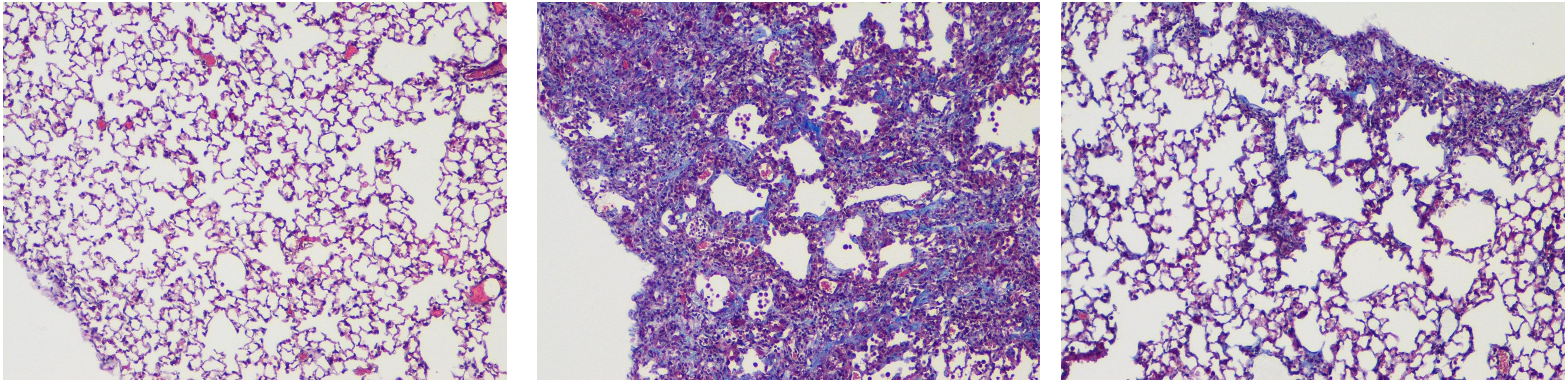

Fig 5. panel I

α-SMA stains:

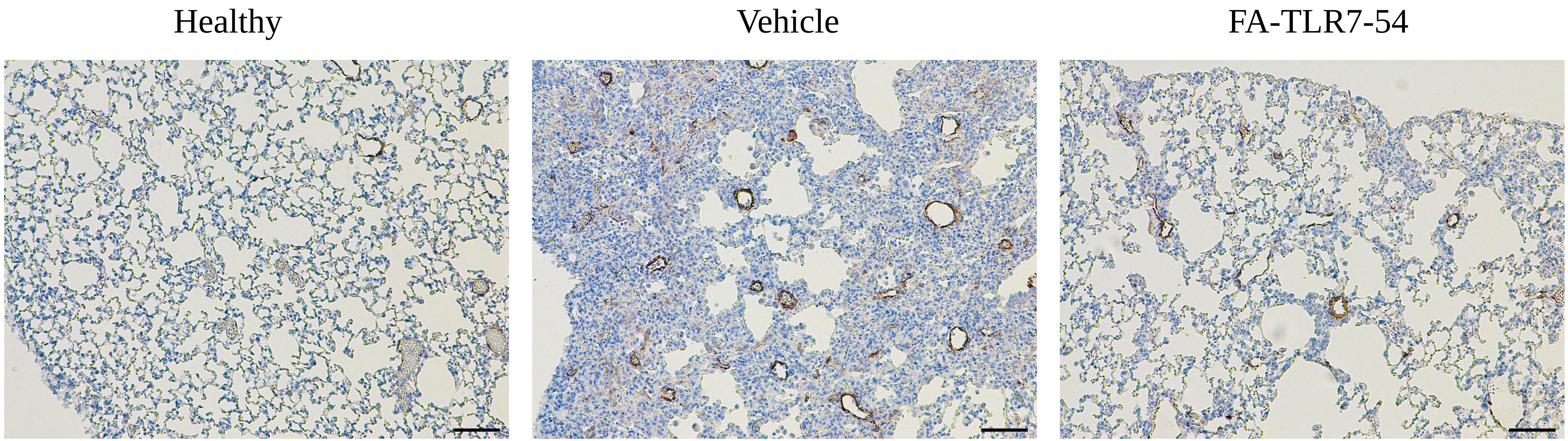

Supplement: Supplementary file 6 — Source Data for Figure 5 [file EMMM-12-e12034-s004.pdf]
