# Supplementary material for: Reprogramming of profibrotic macrophages for treatment of bleomycin‐induced pulmonary fibrosis
Source: EMBO Mol Med. 2020 Jun 29;12(8):e12034. doi: 10.15252/emmm.202012034 (PMC7411553; doi:10.15252/emmm.202012034)

**Fig 6. panel D**

Healthy lungs

H&E

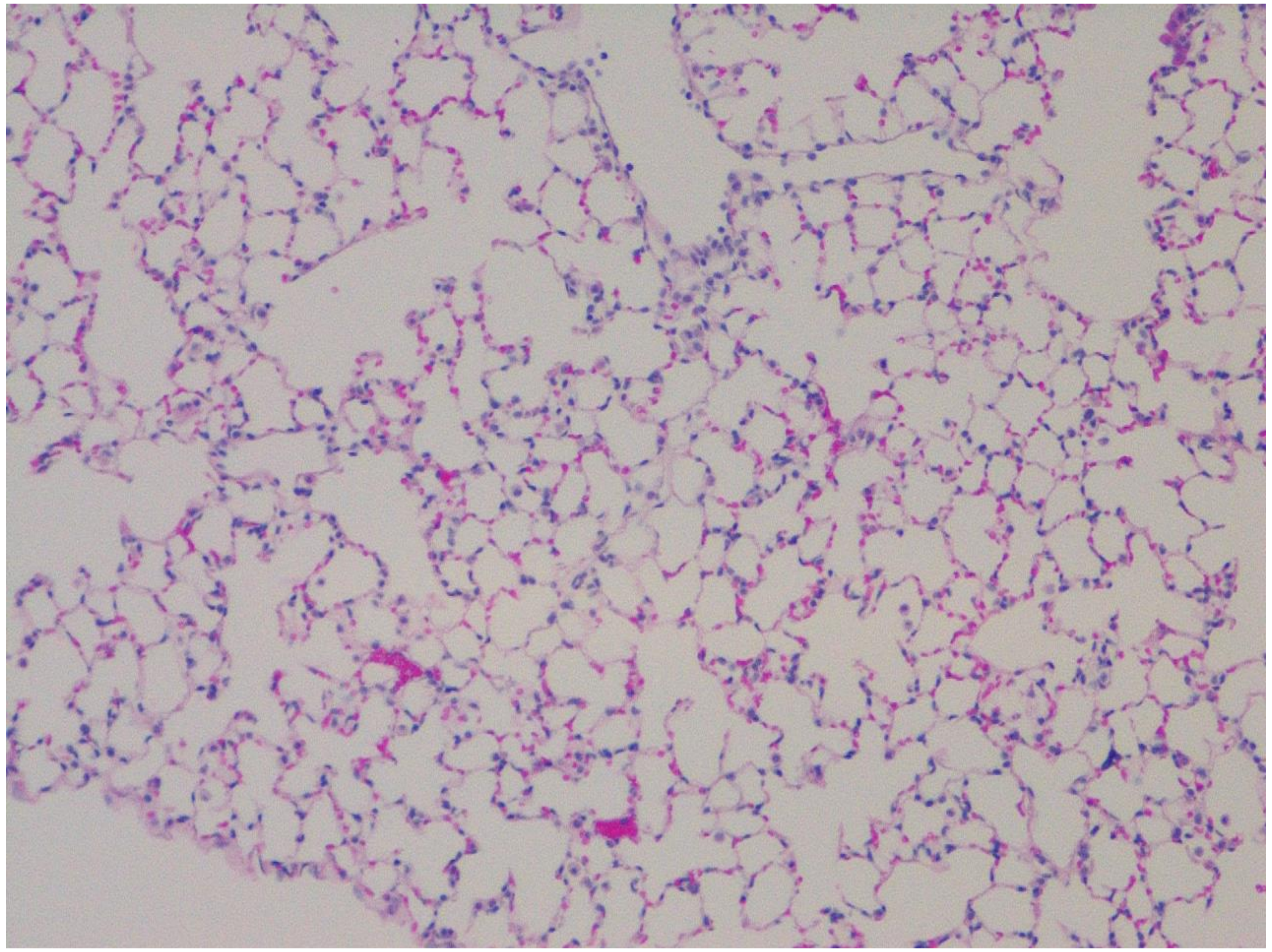

Trichrome

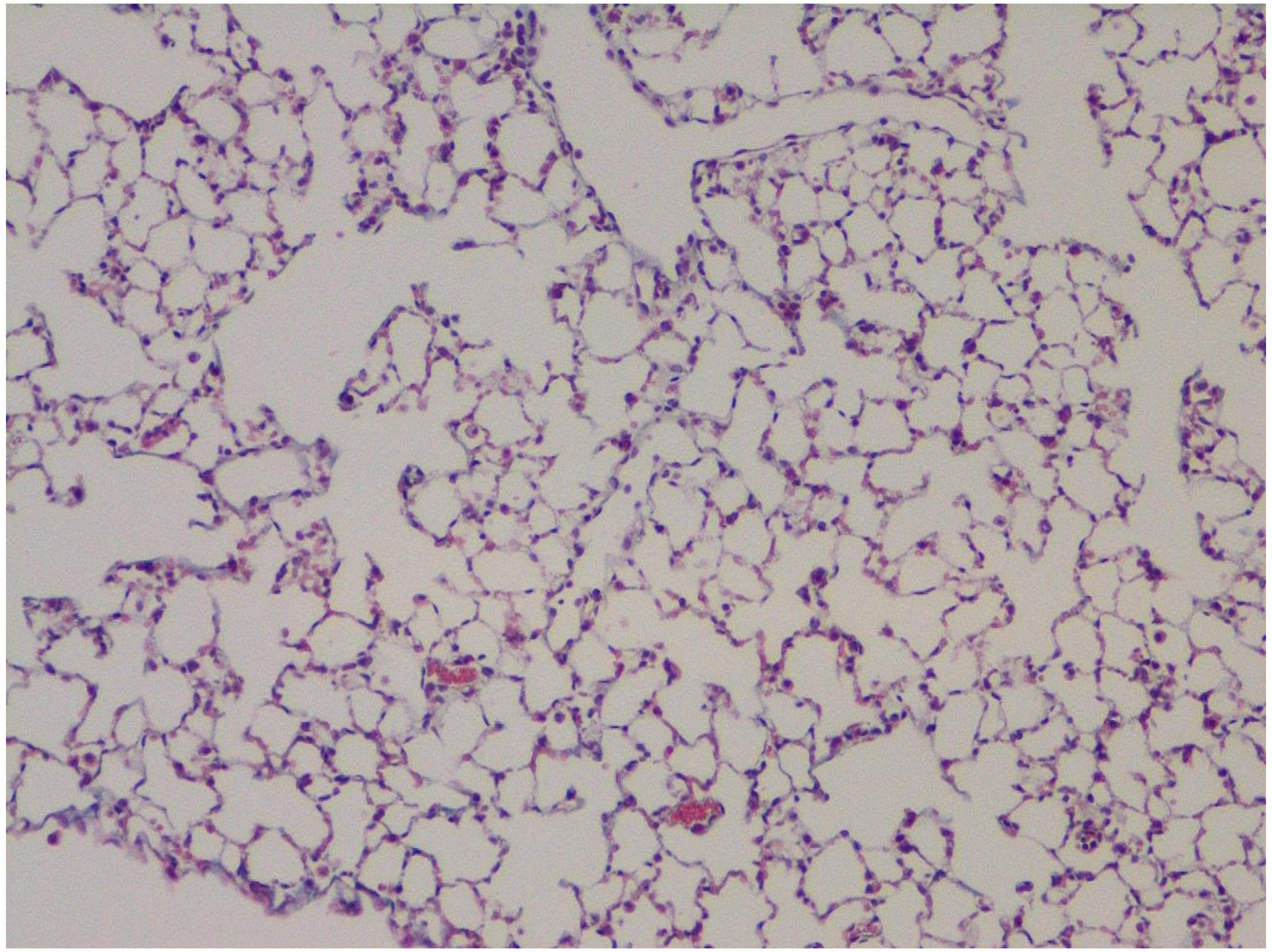

**Fig 6. panel D**

Vehicle treated fibrotic lungs

H&E

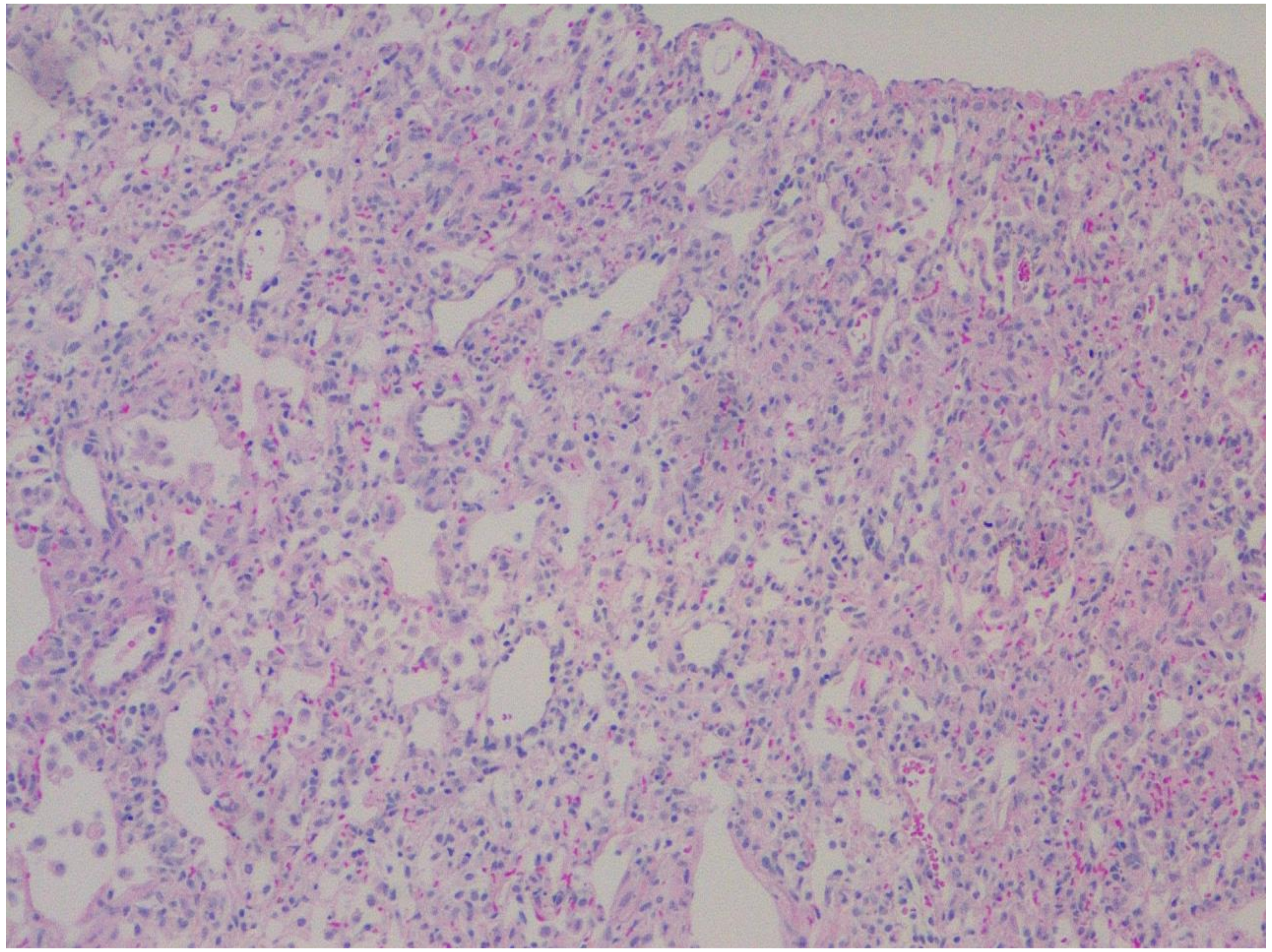

Trichrome

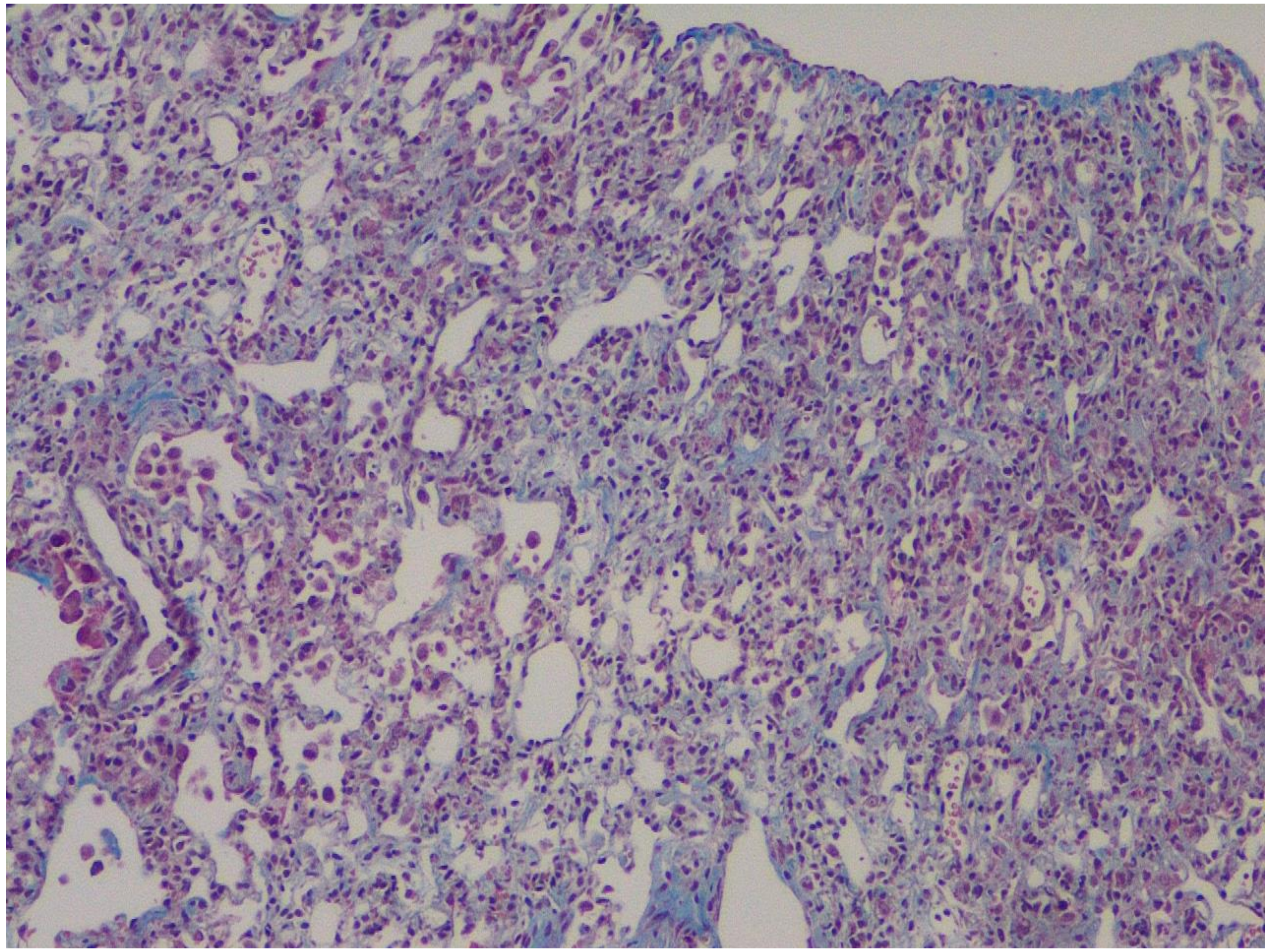

**Fig 6. panel D**

3 nmol FA-TLR7-54 treated lungs

H&E

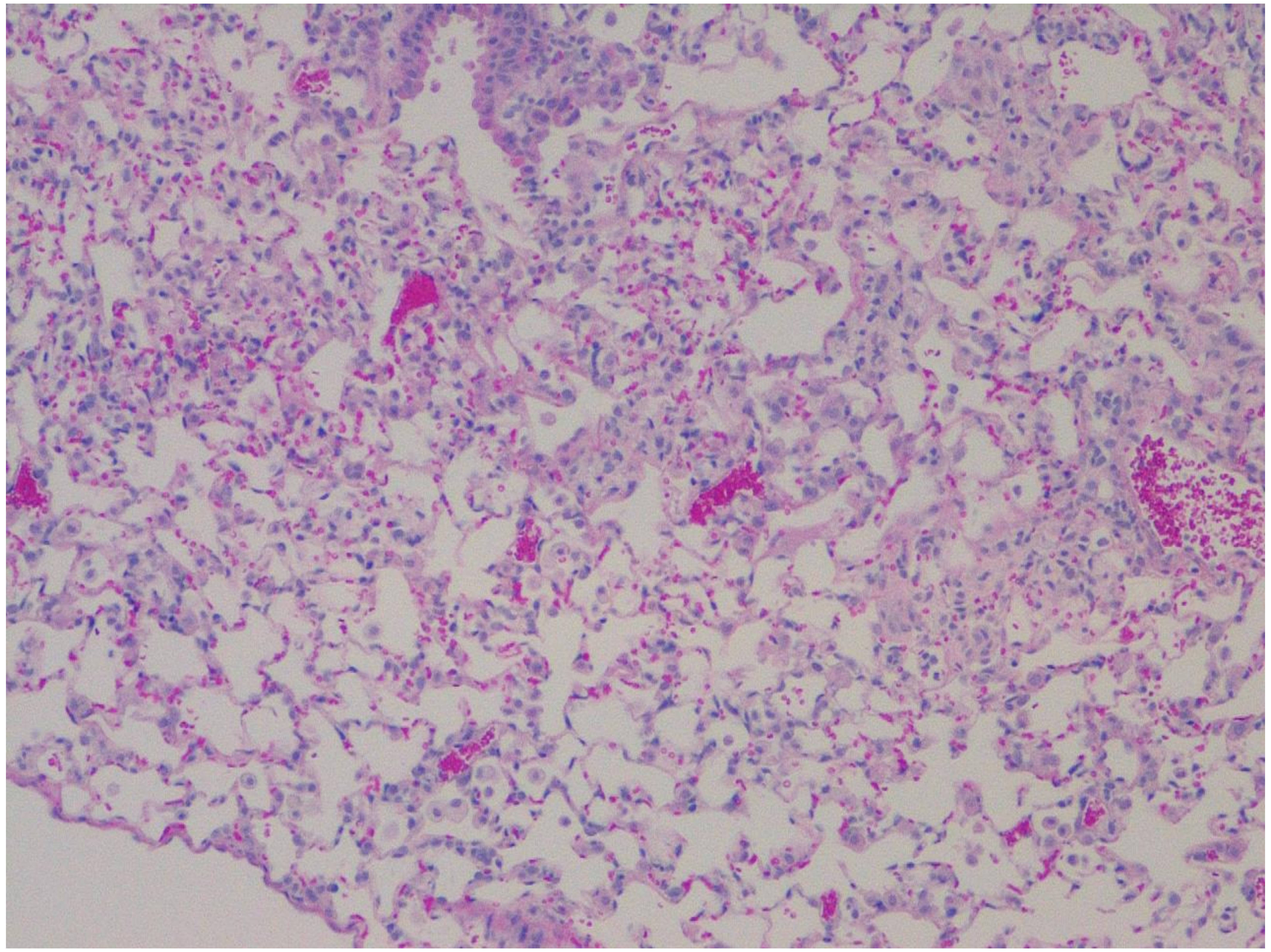

Trichrome

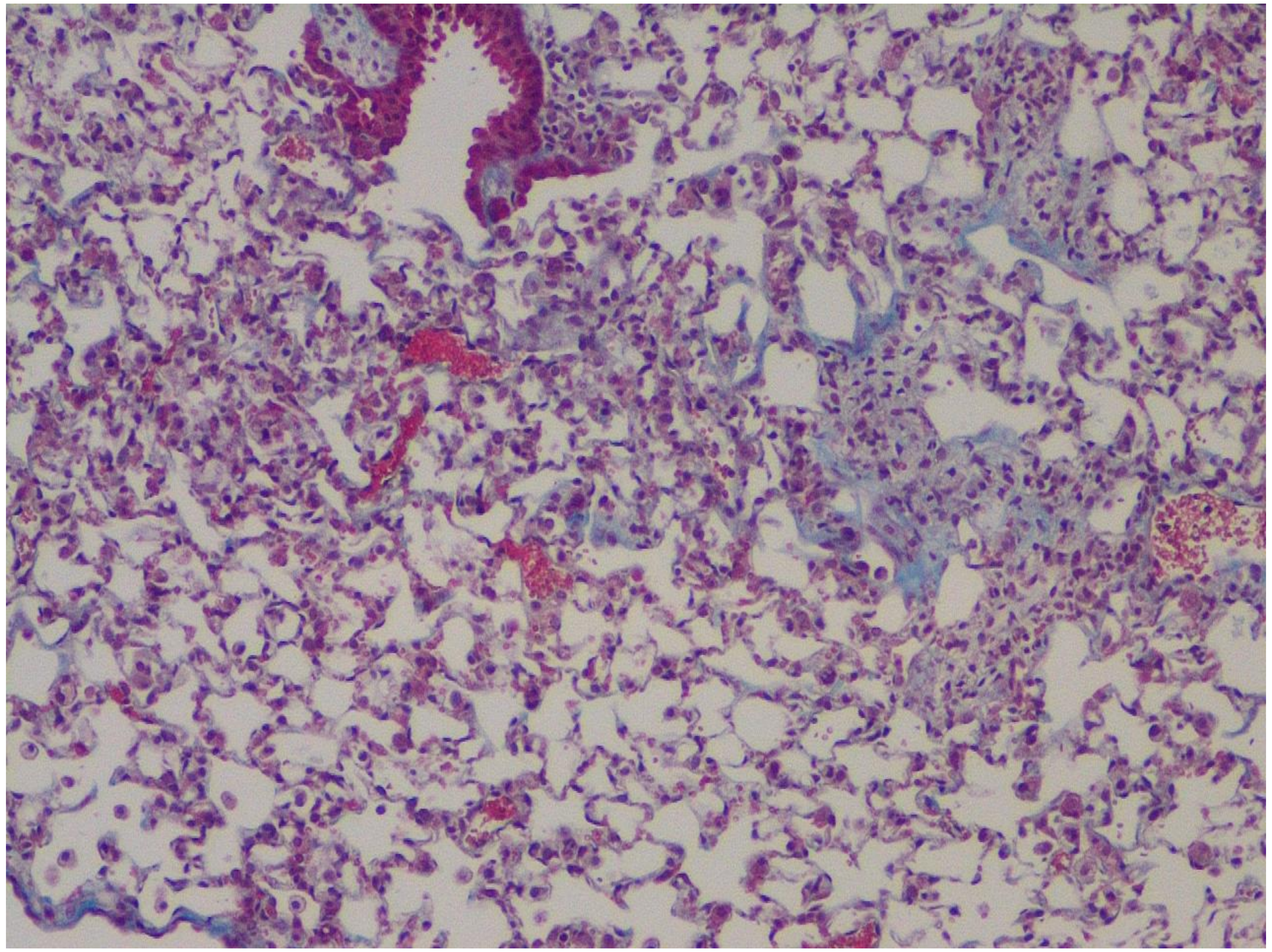

**Fig 6. panel D**

10 nmol FA-TLR7-54 treated lungs

H&E

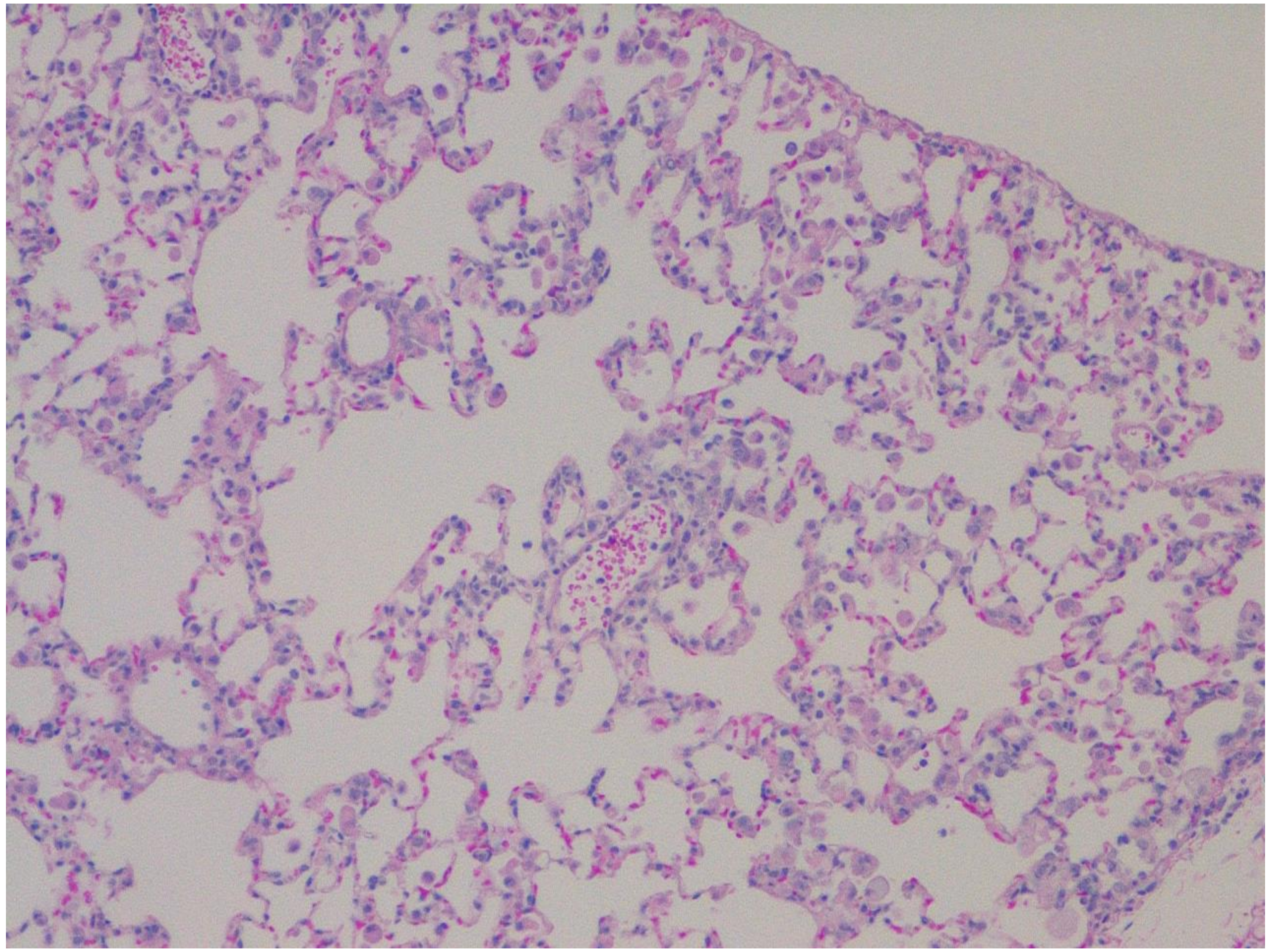

Trichrome

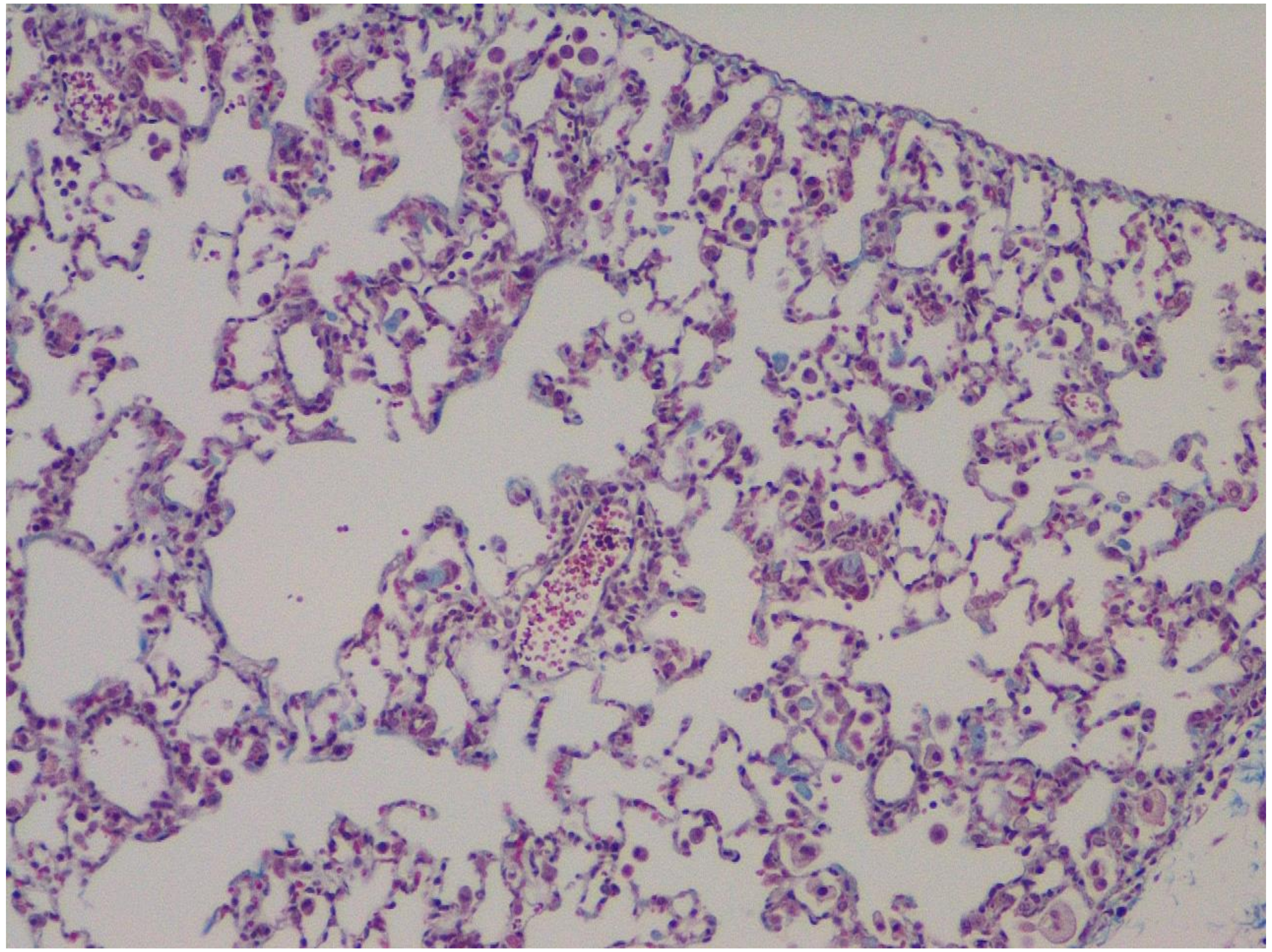

Supplement: Supplementary file 7 — Source Data for Figure 6 [file EMMM-12-e12034-s005.pdf]
